# Catalysis in Chemical Modification of Proteins

**DOI:** 10.1002/cctc.202402125

**Published:** 2025-04-07

**Authors:** Seiya Ishizawa, Koki Fujimura, Kounosuke Oisaki, Shinichi Sato, Jun Ohata

**Affiliations:** a Department of Chemistry, North Carolina State University, Raleigh, North Carolina 27695, USA; b Interdisciplinary Research Center for Catalytic Chemistry (IRC3), National Institute of Advanced Industrial Science and Technology (AIST), Tsukuba, Ibaraki 305-8565, Japan; c Research Fellowship for Young Scientists, Japan Society for the Promotion of Science (JSPS), Tokyo 102-0083, Japan; d Open Innovation Laboratory for Food and Medicinal Resource Engineering (FoodMed-OIL), National Institute of Advanced Industrial Science and Technology (AIST), Tsukuba, Ibaraki 305-0821, Japan; e Frontier Research Institute for Interdisciplinary Sciences, Tohoku University, Sendai, Miyagi 980-8578, Japan; f Graduate School of Life Sciences, Tohoku University, Sendai, Miyagi 980-8577, Japan

**Keywords:** Bioorganic chemistry, Protein modifications, Proteins

## Abstract

Advancement of catalytic transformations in traditional synthetic organic chemistry have made significant impact on development of novel bioconjugation technologies. While a wide range of applications have become possible through catalytic protein bioconjugation approaches, there has been a lack of literature collectively reviewing advances of chemical modification of proteins through the lens of catalysis. This review article is focused on design principles and chemical strategies of nonenzymatic catalysis for targeting natural protein substrates by identifying seven catalysis patterns as organizing topics: electrocatalysis, photocatalysis, metal catalysis, acid catalysis, organocatalysis, supramolecular catalysis, and heterogeneous catalysis. Many literature examples demonstrated possibility of simple translation of small molecule-based catalysis into protein bioconjugation methodologies, whereas others demonstrated unique approaches such as dual catalytic systems and polypeptide structure-specific catalysis design. With a series of successful examples, the survey of catalytic approaches for protein bioconjugation also highlighted the remaining challenges and potential future directions of the area of catalytic bioconjugation.

## 1. Introduction

As the growth of synthetic organic chemistry field was tremendously enabled by various forms of catalytic principles, modern protein bioconjugation strategies also rely heavily on the power of catalysis for fundamental development and various applications. Catalytic transformations have revolutionized industries of small molecule synthesis over the century. Because there has been a growing interest in chemically-modified proteins as renewable resources and materials (e.g., batteries,^[1]^ supercapacitors,^[2]^ antibacterial materials,^[3]^ and enzymes^[4,5]^), catalytic chemical modification of proteins would hold great promise for industrial production of such useful bioconjugates to address the global challenges regarding energy and environment.^[6]^ In addition to the potential improvement of production efficacy of bioconjugates, catalytic platforms can also be leveraged for site-specific modification of proteins for production of therapeutically important agents such as antibody–drug conjugates.^[7]^ Furthermore, a range of chemical tools in biochemistry and chemical biology fields is predicated on catalytic systems enabling target-specific labeling in living systems.^[8]^ Those protein modification applications have been achieved by addressing challenges of protein-targeting catalysis such as aqueous environments, low reaction concentrations (often nM to μM), catalyst deactivation by protein functional groups, and mild reaction conditions (e.g., 37 °C or rt). Despite the substantial development of protein bioconjugation through catalysis, systematic analysis of catalytic bioconjugation has been simply lacking to date, even though numerous reports reviewed the advances of protein bioconjugation either in broader fashions including noncatalytic systems^[9–11]^ or with emphasis on specific catalysis types such as transition-metal catalysis.^[12,13]^

**Figure 1.**
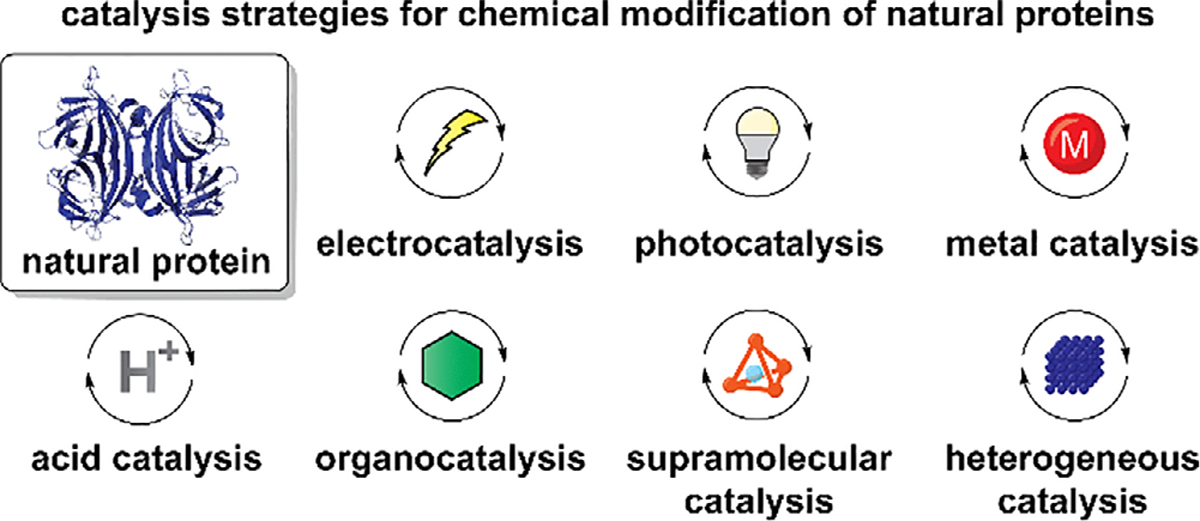
Seven catalysis types for protein bioconjugation discussed in this review article.

This review article is focused on impact of design, selection, and applications of catalysts on protein bioconjugation purposes and categorized into seven types of catalytic transformation mechanisms. The scope of the review article is chemical modification of natural proteins comprised of 20 canonical amino acids, particularly methods without genetic engineering. Methods that can be applicable at a protein level are primarily discussed, and relevant work has been chosen based on arbitrary molecular weight cutoff of 5,000 Da. Importantly, the main focus is on catalytic strategies rather than residue-specific organization found in many existing protein modification review papers.^[14–16]^ Nonetheless, recognizing the practical importance of a list of catalytic bioconjugation methods organized by target amino acid residues and other factors, we included an excel file containing lists of the discussed literature as Supporting Information, in which readers can sort and analyze the collection of papers with a parameter of interest. Among the diverse catalytic systems, seven catalytic mechanisms were used for organization of the article (Figure 1 and Table 1) as the catalysis types are serving as common organizing topics of major catalysis-focused journals such as *Nature Catalysis*, *ACS Catalysis*, and *ChemCatChem*. Although, strictly speaking, only catalytic amounts (i.e., less than the amounts of reactants) should be ideally used for a given catalysis system, bioconjugation processes that are mechanistically catalytic even with the necessary use of excess amounts of a catalyst have been included in this present review. Though bioconjugation often indicates bond formation for functionalization purposes, catalytic bond cleavage reactions were also discussed. Because the focal point of the article is nonenzymatic processes, we refer readers to enzymatic bioconjugation-specific reviews.^[17,18]^

## 2. Electrocatalysis

In this section, we categorize protein modification mediated by electrocatalysts into three types based on their reaction patterns: (1) protein modification through aromatic electrophilic substitution, (2) protein modification via radical addition, and (3) protein modification by nucleophilic substitution.

Electrochemical synthesis, known for its low waste, high selectivity, and mild reaction conditions, has recently attracted attention as a clean approach for small molecule conversions.^[19–21]^ Because of these advantages, electrochemical synthesis has been studied as a method for direct late-stage modification of complex compounds.^[22]^ The potentials make it a promising technique for chemical modification of proteins, which often contain numerous reactive functional groups and can be challenging to purify from a reaction mixture with excess labeling reagents. In Table 2, experimental medium redox potential values of redox active amino acids are shown, and these values are important guidelines for designing electrocatalytic reactions.^[23]^

At a peptide level, electrochemical synthesis offers two methodologies for modification. The first method involves generating an active electron acceptor from a stable precursor via electrode oxidation, which then modifies residues in a peptide through electrophilic substitution reactions. The second method directly generates radical species by single-electron oxidation of a specific functional group in a peptide, which are subsequently captured under suitable reaction conditions to produce a modified peptide. In terms of application to protein modification, the first method is currently applicable to natural proteins or long-chain peptides,^[24,25]^ while the second is limited to peptides with up to ten residues^[26,27]^ or proteins including unnatural 5-hydroxytryptophan residues.^[28]^ Therefore, this review focuses only on the first method through electrode oxidation. However, it is worth noting that in this approach, the modified residues themselves may also undergo electrode oxidation as the reaction progresses.

### 2.1. Protein Modification by Electrophilic Aromatic Substitution

**Scheme 1.**
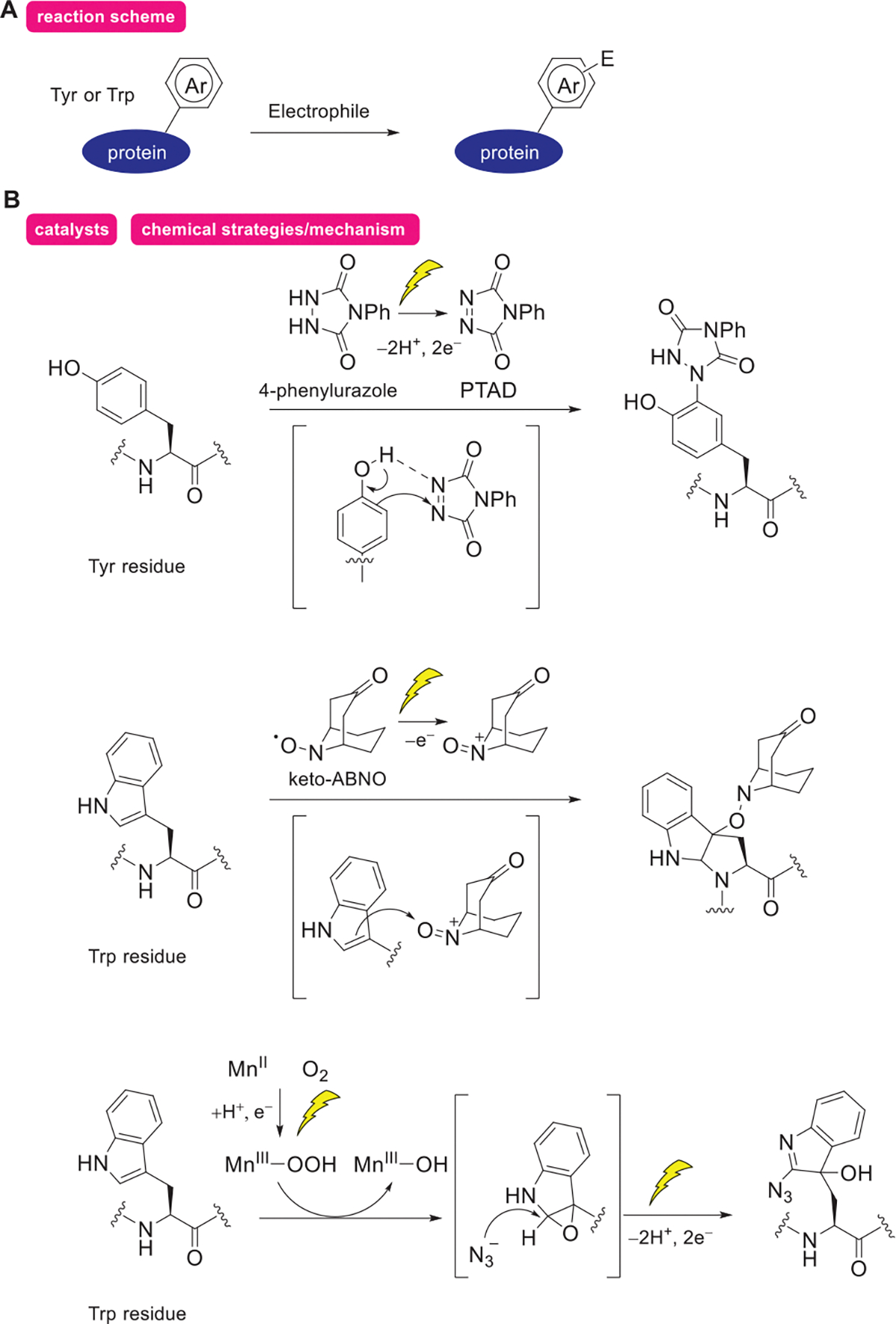
Electrochemical protein modification through electrophilic aromatic substitution: (A) General reaction scheme. (B) Catalysis mechanism of Tyr-ene reaction of 4-phenyl-3*H*-1,2,4-triazole-3,5(4*H*)-diones (PTAD, top), Trp modification by oxoammonium anchors (middle), and Trp modification by epoxidation (bottom).

In this subsection, we introduce a method for protein modification using electrophilic species generated by electrochemical reactions as reactive anchors (Scheme 1A). By applying an appropriate potential specific to the anchor precursor, active anchor sites are oxidatively generated in the reaction system, while preserving the functional groups of the protein. These electrophilic anchor sites modify proteins via electrophilic aromatic substitution reactions at Tyr and Trp residues.

The Tyr-ene reaction of 4-phenyl-3*H*-1,2,4-triazole-3,5(4*H*)-diones (PTAD) and Tyr residues has been extensively studied by Barbas and coworkers,^[29–31]^ and is regarded as a promising approach for protein modification. However, the oxidative generation of PTAD from 4-phenylurazole in such systems is not orthogonal to the various functional groups of proteins. Furthermore, competing side reactions, such as hydrolysis of PTAD, restrict its applicability in a broad context. In 2018, Gouin and coworkers reported the first protein modification employing electrochemical techniques.^[24]^ This method exploits the low redox potential of 4-phenylurazole (+0.36 V vs SCE), enabling rapid Tyr-ene reactions while generating active PTAD species in situ, without oxidizing the aromatic rings of the protein (>0.45 V, Scheme 1B). Li et al. reported that modifying PTAD with azide groups enables protein labeling and detection via a click reaction,^[32]^ which is expected to be further developed as a method for introducing functionality. Another example comes from Heptinstall and coworkers, who reported modifications of protein Tyr residues via iodination or nitration.^[33–35]^ These modifications are achieved through the electrochemical oxidation of KI or NaNO_2_, which activates the reagents.

Trp residues can also be modified by aromatic electrophilic substitution reactions under appropriate conditions (Scheme 1B).^[25]^ The first example was reported by Kanai and coworkers. In this approach, Trp residues are selectively modified by oxoammonium anchors generated by the electrode oxidation of the stable organoradical (keto-ABNO). The redox potential of keto-ABNO (+0.8 V, vs Ag/AgCl) is lowered when it complexes with Trp residues (+0.68 V), enabling functional group tolerance and selective modification of Trp residues. As another example, in 2025, Weng and coworkers reported the modification of peptides up to 31 residues (GLP-1) via oxidation of the indole side chain of Trp residues, using Mn^III^-peroxo species electrochemically generated from molecular oxygen and Mn^II^ as the active species.^[36]^ Unlike general aromatic electrophilic substitution reactions, this method involves the formation of an epoxide on the indole ring as a key step. However, it is also introduced here because it follows a pathway in which two electrons on the aromatic ring react with an electrophilic reagent in a single step.

### 2.2. Protein Modification by Radical Addition

**Scheme 2.**
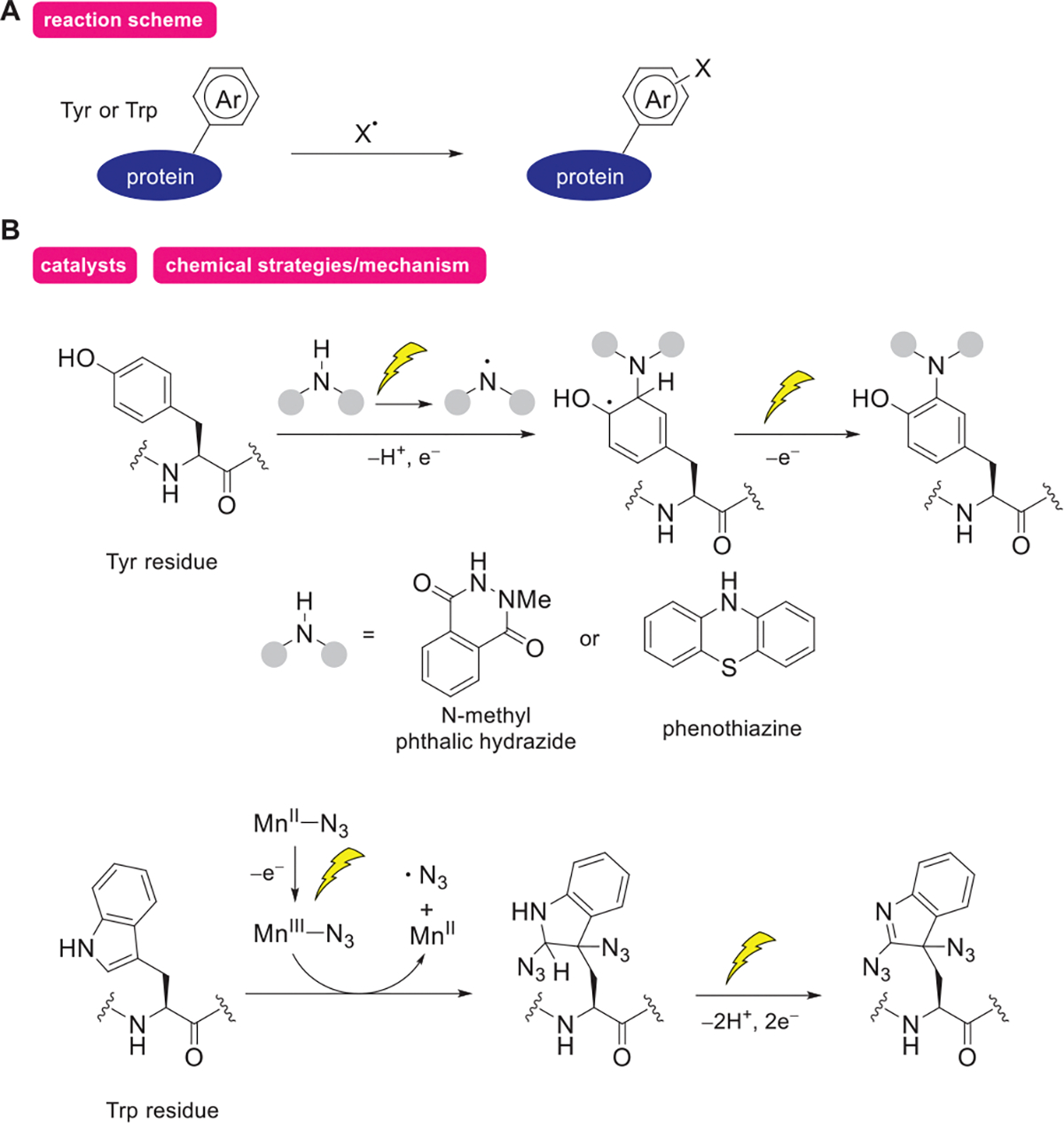
Electrochemical protein modification through radical addition: (A) General reaction scheme. (B) Catalysis mechanism of Tyr-ene reaction in one-electron transfer conditions (top), and Trp modification by azidyl radicals.

In addition to the electrophilic reagents discussed in the previous subsection, electrochemical methods utilizing radical species as anchors have been explored for protein modification (Scheme 2A). In this approach, proteins are modified by radicals generated through one-electron oxidation of anchor precursors at the electrode surface. Radical reactions have been a powerful tool for protein modification because the approach can proceed efficiently in aqueous environments and exhibit low reactivity with polar functional groups.

The Tyr-ene reaction shown in the previous subsection can also proceed under one-electron transfer conditions when 1-methyl-4-phenylurazole or *N*-methyl phthalic hydrazide is used instead of 4-phenylurazole (Scheme 2B).^[37–39]^ In 2020, Nakamura and coworkers reported that nitrogen-centered radicals generated by electrode oxidation of *N*-methyl phthalic hydrazide can modify Tyr residues on variety of proteins.^[38]^ Similarly, Lei and coworkers reported that nitrogen-centered radicals generated by electrochemical oxidation of phenothiazine are also effective for modifying Tyr residues.^[40]^

In 2022, Weng and coworkers introduced a modification method targeting Trp residues using azidyl radicals (N_3_, Scheme 2B). These radicals add to indole side chain, enabling the modification of peptides with 20 or more residues.^[41]^ Azidyl radicals are generated electrochemically from Mn^II^-N_3_, accompanied with C═N double bond formation. Chiang and coworkers subsequently reported that trifluoromethyl radicals (CF_3_·) or thiophenoxy radicals (PhS·) are also applicable to modify Trp residues.^[42,43]^

The methods described in the above two subsections are notable for specifically targeting Tyr or Trp residues for protein modification. These residues are appealing targets because of their low surface exposure, the controlled nature of their modification reactions, and the minimal impact on post-modified protein structure. However, the conversion of phenol and indole side chains typically requires stringent reaction conditions. Electrochemical methods are noteworthy as the strategies enable efficient modification of Tyr and Trp residues under mild conditions, serving as a powerful tool for protein modification.

### 2.3. Protein Modification by Nucleophilic Addition or Substitution

**Scheme 3.**
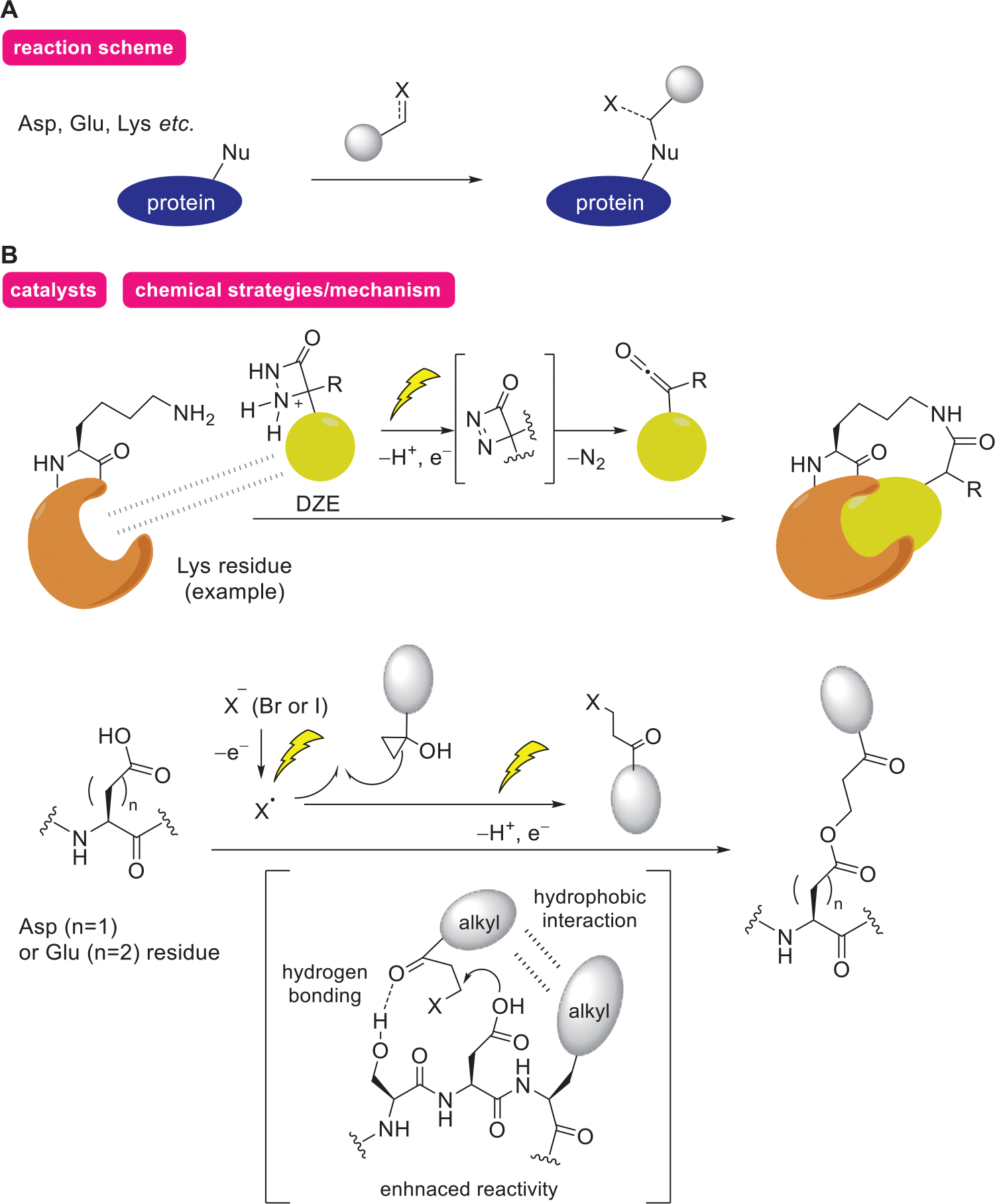
Electrochemical protein modification by nucleophilic addition or substitution: (A) General reaction scheme. (B) Catalysis mechanism of addition to ketene (top), and alkylation of carboxy group.

Reactive species generated by electrolytic reactions can also react with nucleophiles other than aromatic rings, as discussed in this subsection (Scheme 3). Proteins contain a diverse array of nucleophilic functional groups in addition to electron-rich aromatic rings, making them as valuable targets for protein modification strategy.

In 2023, Baran and coworkers reported that electrooxidation of 1,2-diazetidin-3-one (DZE) generates the electrophilic species ketene, which predominantly modifies Glu, Asp, Lys, and Tyr residues.^[44]^ DZE is assumed to generate a highly strained four-membered ring intermediate upon electrode oxidation, which subsequently releases nitrogen molecules to produce highly active ketene. In addition, selective modification of target proteins has been achieved by incorporating their ligands into DZE derivatives in systems containing multiple proteins.

In 2025, Wang and coworkers found that halogen radicals, generated via electrode oxidation of halide anions, react with cyclopropanol to generate *β*-haloketones. These haloketones then selectively undergo nucleophilic substitution with Glu and Asp residues in proteins.^[45]^ It is speculated that the alkylation reaction proceeds when the haloketones and Glu/Asp residues, which have inherently low nucleophilicity, are brought into proximity. This reaction is facilitated by hydrophobic interactions between the alkyl chains of the ketone and the protein side chains, as well as hydrogen bonding between the hydroxyl groups of Ser/Thr residues and the carbonyl groups of the haloketones. The electrochemical generation of cytotoxic haloketones in the system was found to be applicable to protein modification in living cells.

Compared to modification via aromatic electrophilic substitution, these methods can modify a wider range of amino acid residues, including surface-exposed and polar residues. Although residue-selective modifications are more challenging and may alter the structure and charge of proteins, they offer significant advantages for labeling proteins in complex environments.

## 3. Photocatalysis

In this section, we overview four categories of protein modification based on different activation patterns mediated by photocatalysts: (1) oxidative protein modification through electron transfer to a photocatalyst, (2) protein modification activated by the redox cycle of a photocatalyst, (3) protein modification via ^1^O_2_ generation through energy transfer, and (4) protein modification through the activation of a labeling reagent by energy transfer. Photocatalysis, as a robust strategy for activating small molecules, has emerged at the forefront of organic chemistry, experiencing rapid development throughout the 2010s.^[46–48]^ In these approaches, metal complexes and organic dyes engage in single-electron transfer (SET) reactions or energy transfer reactions with substrates, converting visible light into chemical energy. In traditional chemical reactions, the process generally proceeds in a “thermodynamically downhill” direction, making a reaction drive toward an energetically favorable direction through energy release. In contrast, photocatalytic reactions enable the introduction of light energy from an external source, raising the energy level of reactants to facilitate “thermodynamically uphill” reactions, which are typically more challenging to achieve.^[46–48]^ Recently, an increasing number of studies have focused on protein modification through photocatalysis, reflecting a growing interest in this area. While transformations of biomacromolecules using UV light date back several decades including applications in photoaffinity labeling chemistry,^[49]^ approaches utilizing photocatalysis present the advantage of achieving reaction control with visible light, which is considerably more biocompatible than UV light. Reviews have been published, focusing on various perspectives, such as classification based on structural characteristics of catalysts,^[50]^ the targeted amino acid residues,^[51,52]^ the application of photocatalytic chemistry in diverse fields,^[53]^ and photochemistry on a broad range of proteins, including photo-click reactions, nucleic acid modifications, and photo-uncaging.^[54]^ In this section, on the other hand, we will focus specifically on applications of protein modification based on different reaction/catalysis mechanisms

### 3.1. Oxidative Protein Modification Through Electron Transfer to a Photocatalyst

**Scheme 4.**
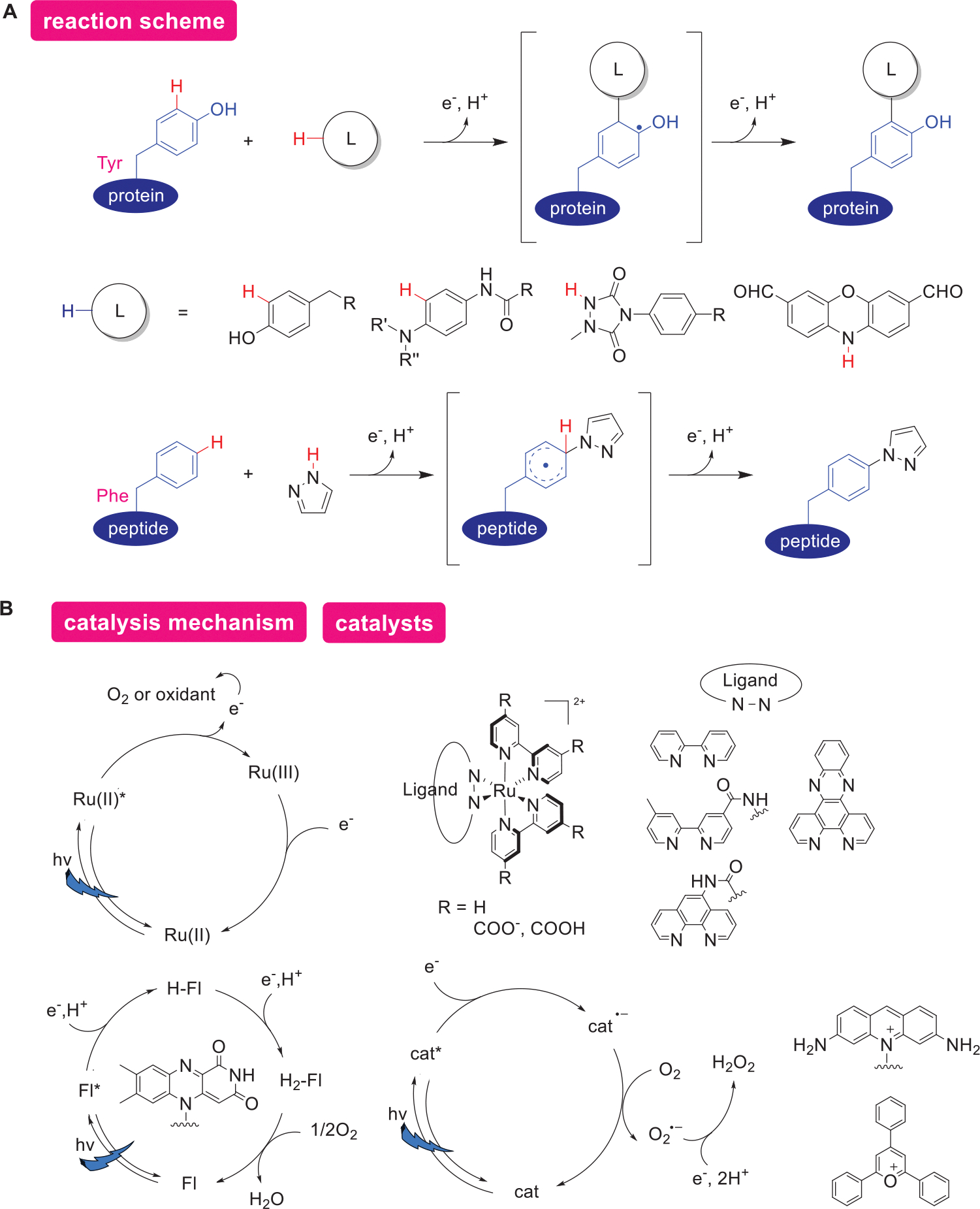
Oxidative protein modification via photocatalysis: (A) Modification of Tyr and Phe through oxidation mediated by SET, along with the structure of the labeling reagents. (B) Structure of photocatalysts and catalytic cycle.

This subsection introduces methods for oxidative protein labeling mediated by electron transfer reactions between photocatalysts and substrates (Scheme 4A,B). In this mechanism, the activated photocatalyst extracts an electron from the substrate, generating a radical species. Based on the oxidation potential of the photocatalyst, radicals can form on both labeling reagents and Tyr residues of the protein. Due to high reactivities of these radical species in general, rapid bond formation often follows the excitation process. With the loss of an additional electron and proton, a reaction proceeds—in which, formally, two electrons and two protons are removed—resulting in the formation of either C–C or C–N bonds.

From the perspective of controlling single-electron transfer reactions, electrochemical methods offer advantages such as precise potential control and the ability to proceed without the need for a catalyst. However, they also present drawbacks, including protein adsorption on the electrode surface,^[55]^ the necessity of adding electrolytes, and significant variability in reproducibility depending on the equipment used. On the other hand, photocatalytic methods require light irradiation and pose challenges in potential control compared to electrochemical approaches, with side reactions involving singlet oxygen and other reactive species sometimes being problematic. Nevertheless, by utilizing light stimuli, which allow for easy control of reaction timing, they enable spatiotemporal control of reactions.

The precise mechanism determining whether the labeling reagent or the Tyr residue on the protein undergoes radicalization, as well as the specific reaction intermediates involved, is still not fully understood and remains open to further investigation. However, in Tyr modifications using tyramide, the radical–radical recombination pathway has been shown to predominate over other pathways between neutral Tyr residue and radical.^[56]^ Additionally, a report indicated that the reaction can proceed even under conditions where the radical species of the labeling reagent is generated electrochemically at potentials that do not directly radicalize Tyr.^[38]^

Several reagents have been reported for Tyr modification, including tyramide, phenylenediamine, *N*-methylurazole, and phenoxazine, while pyrazole has been shown to labelPhe residues. In the catalytic mechanism, for instance, Ru catalysts can be photoexcited, and with the presence of oxidants such as oxygen or ammonium persulfate (APS), transition to a Ru(III) state. This Ru(III) species subsequently oxidizes the substrate via SET as it returns to its ground Ru(II) state. The use of Ru-based photocatalysts has long been known for Tyr–Tyr crosslinking reactions.^[57,58]^ More recently, it has become possible to label proteins by mimicking Tyr residues with tyramide derivatives as labeling reagents. Tyramide conjugated with tags such as biotin (often referred to as “biotin phenol”) enables visualization of labeled proteins and proteomics analysis through enrichment using avidin-beads. Additionally, methods have been explored for switching reactions using complexes with quencher molecules on DNA oligomers.^[59]^

Apart from tyramide, ethylenediamine-type labeling reagents have been utilized for radical modifications of Tyr residues.^[60–64]^ This labeling reagent is also capable of labeling not only Tyr but also Cys, when free Cys residues are located near the catalyst binding site.^[65]^

Additionally, *N*-methylurazole has been developed as a radical-based labeling reagent specifically for labeling Tyr residues in proximity to Ru complexes.^[37,66,67]^ The ligand structure of these catalysts has been examined from multiple perspectives, including the minimization of nonspecific adsorption to proteins,^[63]^ as well as enhancement of ligand binding with proteins.^[67]^ The importance of such proximity effects is described in the supramolecular catalysis section as well.

Covalent bond formation between the catalyst and protein has also been reported, with the Ru(TAP)_2_phen^2+^ complex (TAP = 1,4,5,8-tetraazaphenanthrene; phen = 1,10-phenanthroline) enablingTrp labeling via a SET reaction.^[68]^

For photocatalysts such as flavin, acriflavine, and 2,4,6-triphenylpyrylium (TPT), it is postulated that the excited photocatalyst abstracts an electron from the substrate. Flavin, in particular, can accept two electrons and two protons to achieve its reduced H_2_-Fl state, after which it regenerates its ground state by donating electrons to oxygen. Lumiflavin-based photocatalysts have been applied in selective Tyr modification of proteins using a phenoxazine dialdehyde tag.^[69]^ Flavin-catalyzed Tyr modifications with tyramide have also found applications in analyzing cell-cell interactions by enabling controlled reactivity on cell membrane surfaces.^[70,71]^

Additionally, acriflavine, with its higher cell membrane permeability compared to organometallic complexes, has been adapted for controlling reactions within cells.^[72]^ TPT, known for its high oxidation potential (+2.55 V vs saturated calomel electrode (SCE)),^[73]^ has even been reported to facilitate modifications of Phe residues, which are typically challenging to activate.^[74]^

### 3.2. Protein Modification Activated by the Redox Cycle of a Photocatalyst

**Scheme 5.**
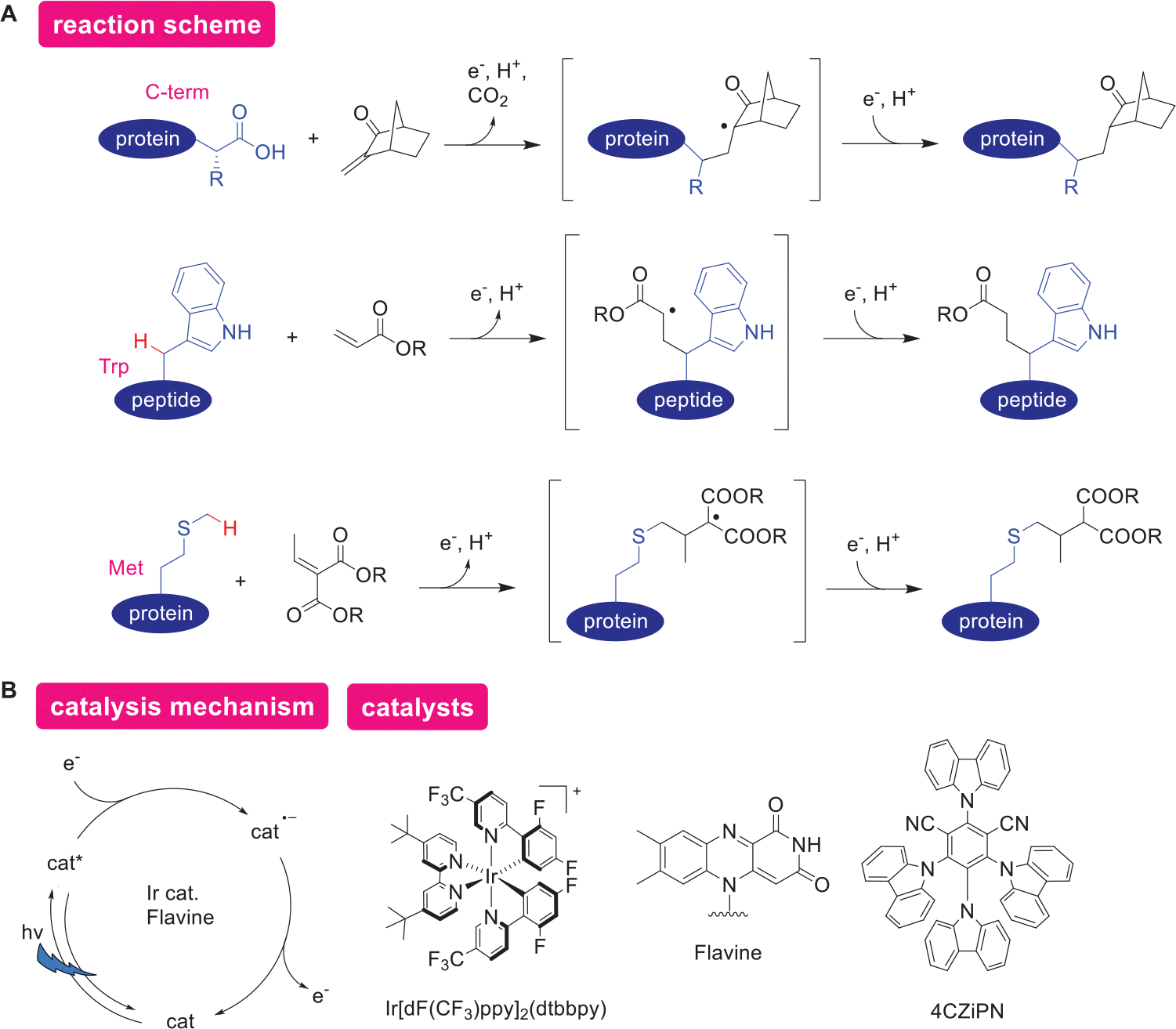
Protein modification via the photoredox catalysis: (A) Reaction mechanism involving the generation of radicals on the protein structure and their capture by labeling reagents. (B) The redox cycle mediated by photocatalysts and the structure of the photocatalysts.

In protein modification through redox processes by photocatalysts, an electron transfers from a substrate to an excited photocatalyst (Scheme 5A,B). This electron transfer results in oxidation of the substrate while the photocatalyst is reduced (cat^−^). Chemical modification of proteins can be achieved at the *α*-position of the C-terminus, the *β*-position of Trp residues, and the methyl group of Met residues as details are described in the following paragraphs. In these instances, it was proposed that radicals formed on the protein are captured by Michael acceptors. Namely, the radical intermediates receive an electron from the photocatalyst in its reduced state, forming bonds as the photocatalyst returns to its ground state.

The C-terminus can be preferentially reduced via a single-electron process at a lower potential compared to the carboxylates of Asp or Glu in proteins (*E*_1/2_^red^: ~1.25 V for Asp, Glu; ~0.95 V for C-terminus vs SCE). The carbon radicals generated from CO_2_ loss can be captured by Michael acceptors such as diethyl ethylidenemalonate and 3-methylene-2-norbornanone. Selective modification of the C-terminus using flavin photocatalysts in various peptides and insulin has been documented.^[75]^ This pioneering study established a fundamental strategy for site-selective photocatalytic bioconjugation at the C-terminus, inspiring the strategy of capturing and modifying radicals generated within protein structures.

For peptide substrates, use of an iridium photocatalyst (Ir[dF(CF_3_)ppy]_2_(dtbbpy)PF_6_, *E*_1/2_^red^: 1.21 V vs SCE) alongside a polyaromatic photocatalyst (4CzIPN, *E*_1/2_^red^ = 1.35 V vs SCE) for C-terminal alkynylation with ethynylbenziodoxolone (EBX) reagents,^[76]^ and conversion of the C-terminus to N,O-acetals for electrophilic activation^[77]^ have been reported. Unique to Trp modification is that labeling the *β*-position of the Trp side chain using Ir[dF(CF_3_)ppy]_2_(dtbbpy)PF_6_ with Michael acceptor modification has been accomplished.^[78]^ In a report of Met modification, lumiflavin (*E*_1/2_^red^ = 1.5 V vs SCE), that is capable of accepting an electron from Met (*E*_pa_ = 1.36 V vs SCE), was utilized, as reduced lumiflavin (HLF^·^) facilitates proton transfer from the Met radical cation (pKa =~3.5), acting as a base (pKa of HLF^·^ = 8.5) and catalyzing the transfer of electrons and protons.^[79]^ The extension of these photocatalysts’ redox cycles for peptides substrates to protein bioconjugation is based on similar mechanisms, necessitating thorough evaluation of reaction conditions to ensure orthogonality and mitigate side reactions with Michael acceptors and residues such as Cys and Lys.

An exceptional example of photocatalytic protein bioconjugation involves using a quinolinone chromophore-based photocatalyst to accelerate the thiol-ene reaction between Cys and terminal olefins through hydrogen-atom transfer catalysis.^[80]^ Activation of electrophilic species employing SET and combinations of SET and hydrogen atom transfer (HAT) for Cys modification^[81]^ andHis modification^[82]^ are also noteworthy. Recently, a photocatalytic Tyr phosphorylation method utilizing the radical Arbuzov reaction has also been reported, in which a tyrosyl radical generated via SET is trapped by a phosphite reagent.^[83]^

### 3.3. Photocatalytic Protein Modification via ^1^O_2_ Generation Through Energy Transfer

**Scheme 6.**
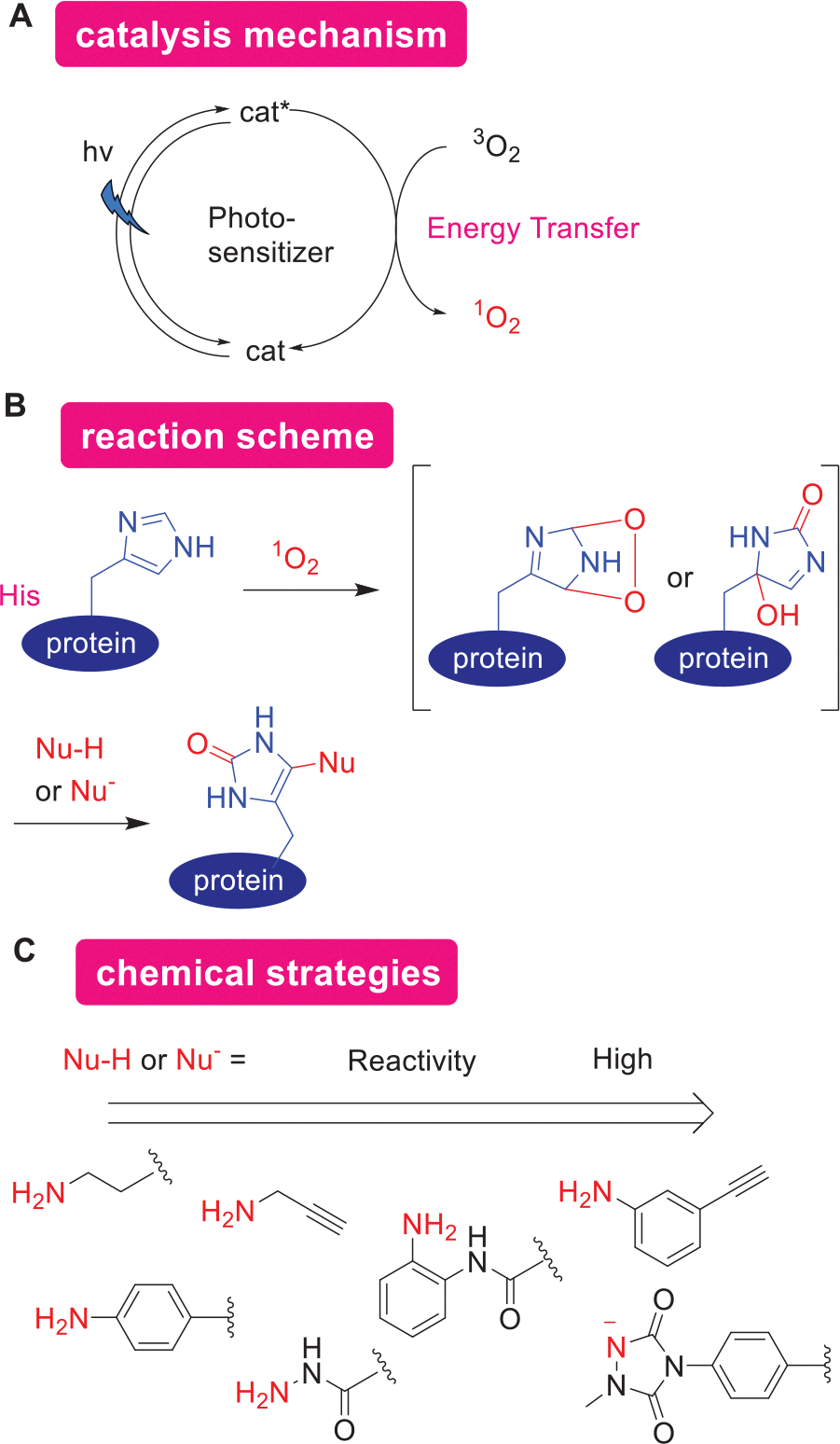
Protein modification utilizing ^1^O_2_ generation from a photocatalyst: (A) Generation of ^1^O_2_ through energy transfer reactions between an excited photosensitizer and oxygen molecules. (B) A technique involving the capture of electrophilic intermediates produced by Diels–Alder reactions between His residues and ^1^O_2_ using nucleophiles. (C) The structure and reactivity of the nucleophiles used in this method.

Distinct from photocatalytic mechanisms that involve a SET, various protein modification techniques have utilized energy transfer mechanisms from an excited catalyst to the substrate. Numerous photo-responsive molecules known as photosensitizers activate molecular oxygen to generate highly reactive singlet oxygen, ^1^O_2_ (Scheme 6A). ^1^O_2_, which has a lifespan of only microseconds in water and limited diffusion,^[84]^ leads to oxidation reactions in close proximity to the photosensitizer. His residues are primary targets for ^1^O_2_-induced oxidation, undergoing Diels–Alder additions that form reactive endoperoxide intermediates on the imidazole rings. Mechanistic studies have shown that these reactions do not proceed through stepwise oxidation and nucleophilic addition (Scheme 6B),^[85,86]^ indicating that the formed reactive species are electrophilic. There was a demand for nucleophiles that can efficiently capture these active species, as such nucleophiles enable various tagging applications. According to a few studies comparing different nucleophiles, 3-ethynylaniline demonstrated higher reactivity compared to other anilines, amines, and phenylhydrazines.^[86]^ Additionally, 1-methyl-4-arylurazole (MAUra), with a pKa of 4.7,^[87]^ predominantly exists in its anionic (N^−^) form at neutral pH, enhancing its nucleophilicity and thereby facilitating the efficient capture of oxidized His.^[85]^

This strategy for protein modification, known as proximity labeling (PL), exploits the proximity-dependent nature of photocatalysis and the brief lifespan of ^1^O_2_. By generating ^1^O_2_ and capturing oxidative intermediates nucleophilically (Scheme 6C), it facilitates a wide range of applications. These include identifying RNA-binding proteins,^[88,89]^ site-selective modification of antibodies,^[85,90]^ controlling reactivity within cells for subcellular proteomic mapping,^[91,92]^ analyzing His on aggregated proteins after catalytic photo-oxygenation,^[93]^ examining metal-binding His,^[86]^ controlling surface reactions for cell–cell interaction studies,^[94,95]^ and interactome analyses in live mouse brains.^[96]^

While this review primarily focuses on protein bioconjugation, it is notable that the concept of using photocatalysts to generate reactive oxygen species, thereby oxidizing and degrading proteins, has been well-established in techniques such as chromophore/fluorophore-assisted laser inactivation (CALI/FALI).^[97–99]^

### 3.4. Protein Modification Through the Photoactivation of a Labeling Reagent by Energy Transfer

**Scheme 7.**
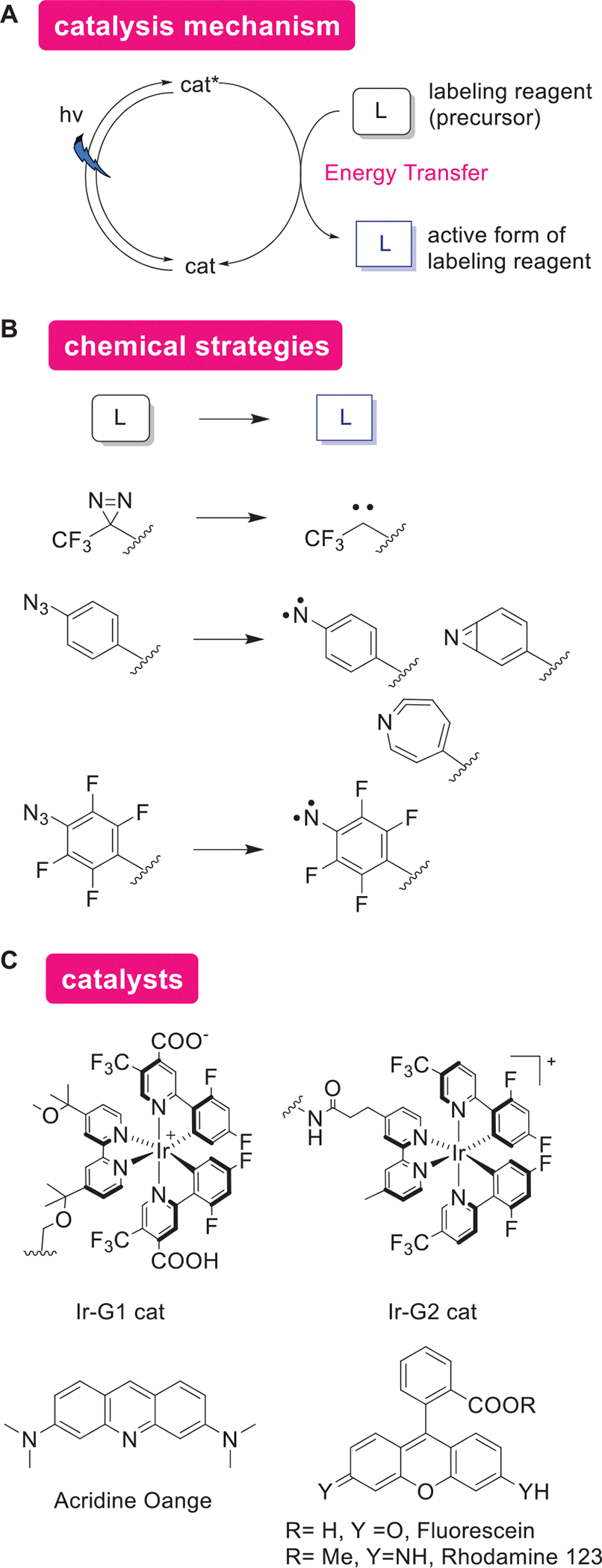
Protein modification utilizing energy transfer from a photocatalyst: (A) Activation of modifiers through energy transfer from an excited photocatalyst. (B) Reactive species that can be generated by this method, capable of labeling various amino acid residues. (C) The structure of the photocatalysts used in this method.

Developments have also been made in methods that transfer energy and activate labeling reagents (Scheme 7A). Such activation could proceed through Dexter energy transfer between an excited Ir-photocatalyst and a diazirine-based labeling reagent, for instance (Scheme 7B,C). This activation of diazirine in the proximity of the catalyst generates carbene, a highly reactive chemical species with a half-life of 2 ns.^[100]^ The photoactivation allows for precise control of protein modification reactions within a tightly restricted area less than 4 nm around the catalyst.^[101]^ The first-generation Ir photocatalyst based on Ir[dF(CF_3_)ppy]_2_(dtbbpy) (Ir-G1 cat) not only produces carbene but also activates arylazide, generating nitrene.^[102]^ This catalytic activity has been applied to analyze protein-protein interactions (PPI) on cell membrane surfaces,^[101]^ ligand binding site mapping,^[103]^ control labeling radius by altering labeling reagents,^[102]^ study binding proteins of sialylated glycoproteins,^[104]^ and dynamic analysis of phagocytic surfaces.^[105]^ Additionally, the second-generation Ir photocatalyst (Ir-G2 cat), which addresses cell membrane permeability issues of the first generation catalysts, has been employed for small molecular compound target identification,^[106]^ analysis of PPI in chromatin proteins,^[107]^ and application to stress granule components in cells.^[108]^

Activation of arylazide has been further explored using organic dyes such as acridine orange, fuorescein, rhodamine 123,^[109]^ a red light-activated osmium photocatalyst,^[110]^ and Sn^IV^ chlorin e6 catalyst.^[111]^ These catalysts function through a SET mechanism rather than energy transfer, involving the reduction of arylazide in the presence of NADH through a stepwise reduction–dissociation–oxidation pathway.

As an alternative method, photocatalytic conversion of arylazide to aniline has been utilized in photo-uncaging techniques. This approach also produces *o*-thioquinone methide, an electrophilic species used for protein modification and subcellular proteomic mapping.^[112]^

## 4. Metal Catalysis

Metal-catalyzed protein modification has been enabled through a series of chemical strategies such as bioorthogonal chemistry and design/utilization of protein-compatible transitionmetal complexes. A number of metal-mediated protein modifications were achieved by the use of noncanonical amino acids. For instance, copper-catalyzed azide-alkyne cycloaddition—a quintessential “click” reaction— has been widely used by introducing an azide or alkyne handle onto proteins through methods such as chemical modification and genetic/metabolic incorporation.^[113,114]^ Similarly, instillation of arylboronic acid or aryliodide allows for Suzuki–Miyaura coupling with a palladium catalyst.^[115–117]^ Olefin metathesis with a Grubbs-type ruthenium complex is also possible by introduction of noncanonical alkene groups with a thioether moiety.^[118–120]^ A large portion of metalmediated modification of canonical amino acid residues, on the other hand, is often a noncatalytic system. Even for catalytic reactions of natural proteins, an excess amount of metal catalysts is necessary likely because of interaction of proteins with metal salts and because of challenges of catalysis in aqueous media. Indeed, there are many catalytic systems that uses organic solvents that are typically not compatible with protein substrates (but peptides).^[121,122]^ Examples described below tackled the challenges of protein modification in aqueous solutions by modulation of the reactivity of a metal catalyst such as the use of a coordinatively saturated complex to avoid undesired interactions with proteins. As there have been multiple review articles about metal-based protein bioconjugation in the past,^[123–126]^ this section will be focused on catalytic aspects of the processes. The comparison of the relative Lewis acidity of different metals was investigated by a fluorescence-based method.^[127,128]^ The toxicity of metal compounds is reported as LD_50_ (oral rat) values.^[129]^ It should be noted that given metal’s toxicities vary by their oxidation states and counter anions.

### 4.1. Metallocarbene/Metallonitrene

**Scheme 8.**
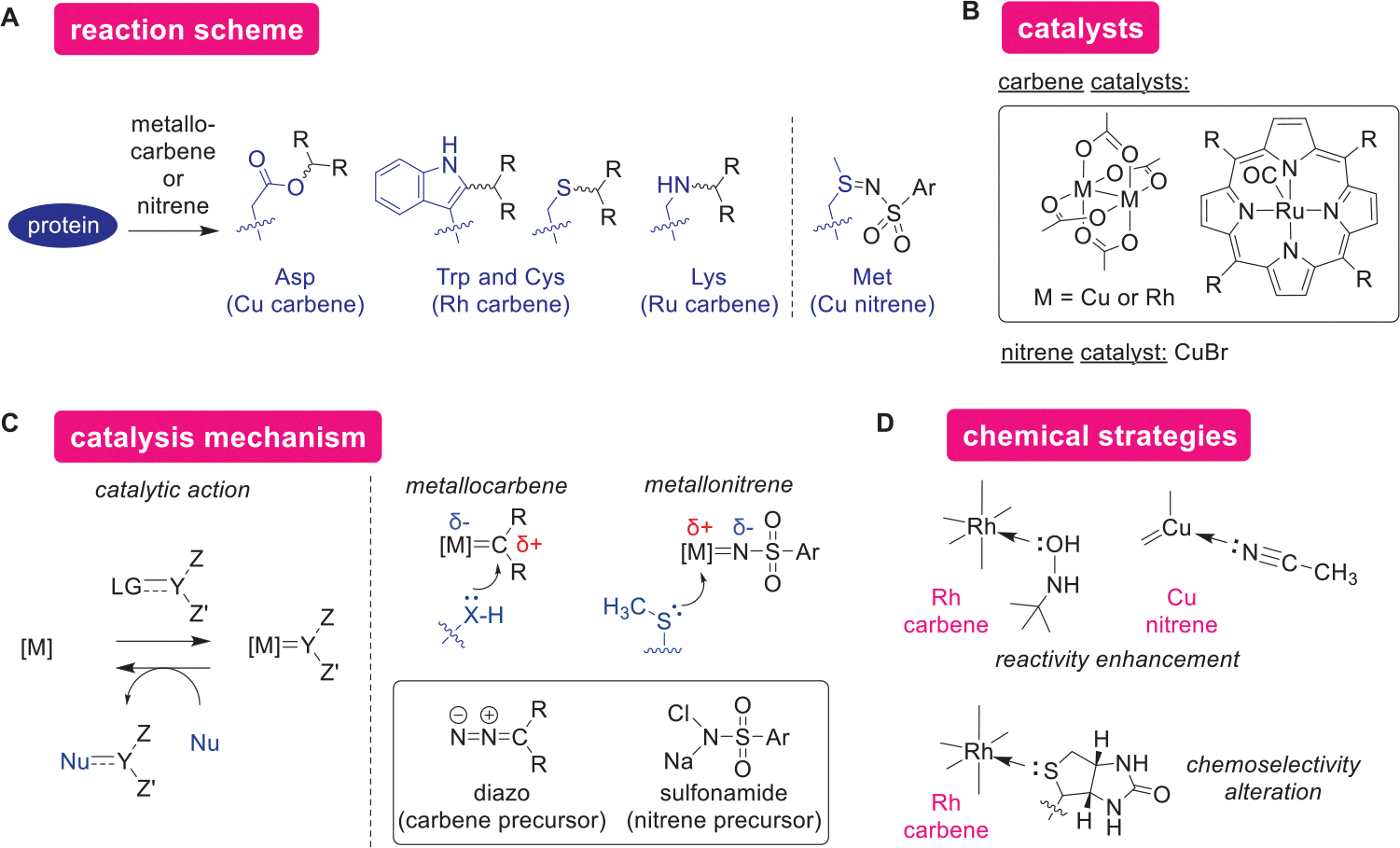
Carbene and nitrene catalysis by copper, rhodium, and ruthenium complexes: (A) General reaction scheme. (B) Chemical structures of catalysts. (C) General depiction of mechanism of actions of catalysts. (D) Coordination of an additional ligand to a metal center as strategies to increase catalytic activity or alter chemoselectivity.

Metallocarbene- and metallonitrene-based catalysis has been widely explored for chemical modification of natural proteins (Scheme 8A–C). The carbene and nitrene chemistry functions with the nucleophilic reactivity of amino acid side chains such as indole (Trp) and amine (Lys) through generation of electrophilic metal species from stable precursor molecules (e.g., diazo and sulfonamide compounds). Copper,^[130,131]^ rhodium,^[132]^ and ruthenium^[133]^ complexes have been shown to be useful for targeting various amino acid residues through metallocarbene intermediates (Scheme 8B). The majority of the metallocarbene-based strategies relied on discrete complexes such as a paddle-wheel rhodium complex and ruthenium porphyrin complex, perhaps increasing the lifetime of the catalysts in aqueous solution in the presence of the nucleophilic biomolecules. Those metal complexes exhibits electrophilic nature, and the reactivity of the carbene complex can be enhanced through coordination of a buffer component to the metal center (Scheme 8D).^[134]^ Such a ligand binding can also cause the alteration of chemoselectivity of dirhodium carbene reactivity toward Trp to Cys by use of a reagent bearing thioether group (i.e., biotin group).^[135]^ The rhodium carbenoid system can be coupled with a proximity-driven strategy to target many amino acid residues other than Trp (see the supramolecular catalysis section).^[136]^ More recently, copper nitrene complex was shown to act as a Met-selective protein modification method, where copper bromide salt and sulfonamide are the precursors of the nitrene complex.^[137]^ The unique chemoselectivity of the nitrene chemistry was ascribed to the thioether reactivity to the electrophilic metal center bound with acetonitrile ligands. As such, there has been a variety of carbene- and nitrene-based metal catalysis for protein modification since the initial report of the Trp-selective carbenoid method as one of the early examples of the modern protein bioconjugation study.^[132]^ In addition to the carbene and nitrene catalysis, a copper-catalyzed azide transfer reaction using sulfonyl azide to alkyl amine groups of proteins should be mentioned here, as the transfer mechanism has similarity to the diazo reagent preparation.^[138]^

### 4.2. Cross-Coupling

**Scheme 9.**
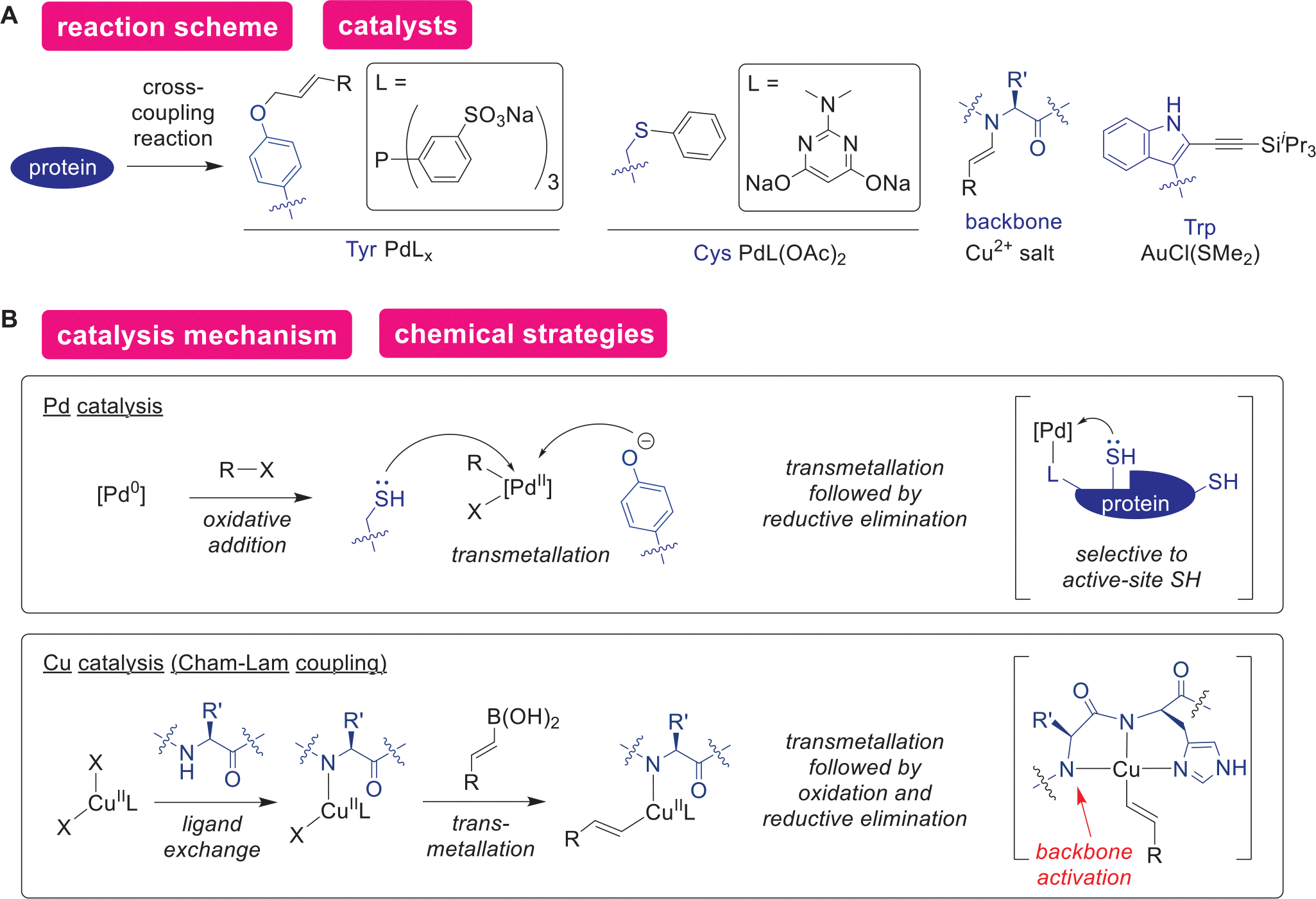
Metal-catalyzed cross-cross coupling reactions: (A) Chemical structures of the reaction products on modified amino acid residues, catalysts, and ligands. (B) Mechanism of actions for the palladium and copper catalysis.

Cross-coupling reactions are another class of metal catalysis that has been one of the focal points in the protein modification field (Scheme 9A,B). As various palladium cross-coupling methods emerged in the realm of synthetic organic chemistry during the past decades,^[139–141]^ the bioconjugation field has also been extensively examining their capabilities and utilities. The fundamental catalytic actions and reaction mechanisms of such cross-coupling bioconjugation approaches follow the same principle as that of the small molecule chemistry (e.g., oxidative addition, transmetallation, and reductive elimination). Often, electrophilic nature of a metal center after oxidative addition has been leveraged for both catalytic and noncatalytic protein bioconjugation strategies.^[142]^ A Tyr-selective palladium-catalyzed approach (Tsuji–Trost coupling) is one of the earliest examples among the palladium-based methods, driven by deprotonation of the phenol group making it a favorable nucleophile at high pH over other amino acid side chains.^[143]^ Another catalysis example is active-site Cys selective through coordination of an auxiliary ligand to the palladium center.^[144]^ Other metal sources can be utilized for cross-coupling reactions of proteins including copper catalysis with use of boronate compounds as transmetallation reagents that modifies the amide backbone N–H group (Chan–Lam coupling).^[145]^ The original report by Ball and coworkers made use of a copper binding motif with a His residue—that is akin to known metal-binding peptide sequence called amino terminal copper and nickel (ATCUN) motif— to activate the backbone N–H and to facilitate the reductive elimination between the amide and boronate-derived group.^[146]^ Recently, use of a different solvent^[147]^ and different amino acid binding patterns^[148]^ have been shown to achieve similar chemistry without aids of the His residues as well. Gold would be another metal source that allows for cross-coupling reactions on proteins targeting a Trp residue in aqueous acetonitrile solution.^[149]^ While this specific chemistry is the sole example of cross-coupling-based gold catalysis, several noncatalytic Au bioconjugation approaches have been reported to date,^[150,151]^ and together with its relatively lower toxicity compared to other transition metals,^[152,153]^ development of gold catalysis may be merely in a nascent state for potential growth.

### 4.3. Metal Hydride Reduction

**Scheme 10.**
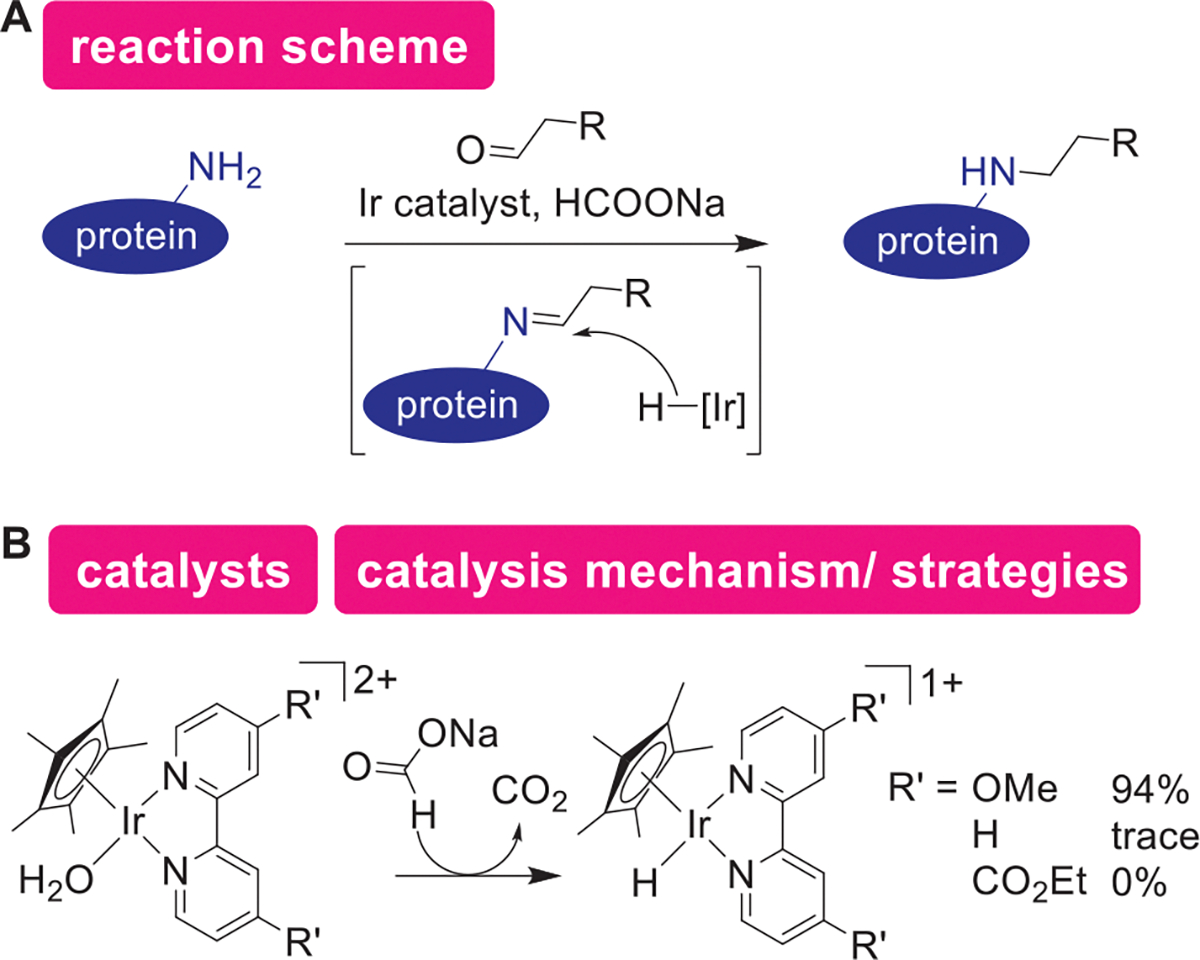
Iridium-catalyzed reductive alkylation/amination: (A) General reaction scheme. (B) The structure of the procatalyst (left) and active catalyst after reduction with sodium formate (right).

While redox processes are often utilized for protein modification processes, iridium hydride-based catalytic reductive alkylation remains the sole example of the catalytic reduction for protein bioconjugation purposes in this metal catalysis section (Scheme 10A,B).^[154]^ Analogous to the traditional protein modification strategy using aldehyde and cyanoborohydride reagents,^[155]^ the iridium-based hydride reduction would occur through the Schiff base formation from the protein amines followed by the reduction of the Schiff base to amine. The catalytic system reported by Francis and coworkers utilized activation of a pro-catalyst, water-soluble iridium Cp* complex through reduction with sodium formate (Scheme 10B). Electronrich bipyridine ligands were found to be particularly effective, which perhaps is an indication of importance of nucleophilicity of the metal hydride species. While this iridium catalysis has been used in other reports, there has not been other metal-catalyzed reductive approaches developed for protein bioconjugation. The lack of the development may be due to the challenges of retention of reduction-sensitive S–S bonds in proteins, although mild and/or bulky reductants such as ascorbate^[156,157]^ and triarylphosphine^[158]^ have been successfully utilized in the protein labeling strategies. Reduction-based metal catalysis may grow dramatically for protein modification fields, as reductive metal catalysis (e.g., metal hydride chemistry) has been useful in many chemical biology applications.^[159,160]^

### 4.4. Oxidative Coupling Through Metal Catalysis

**Scheme 11.**
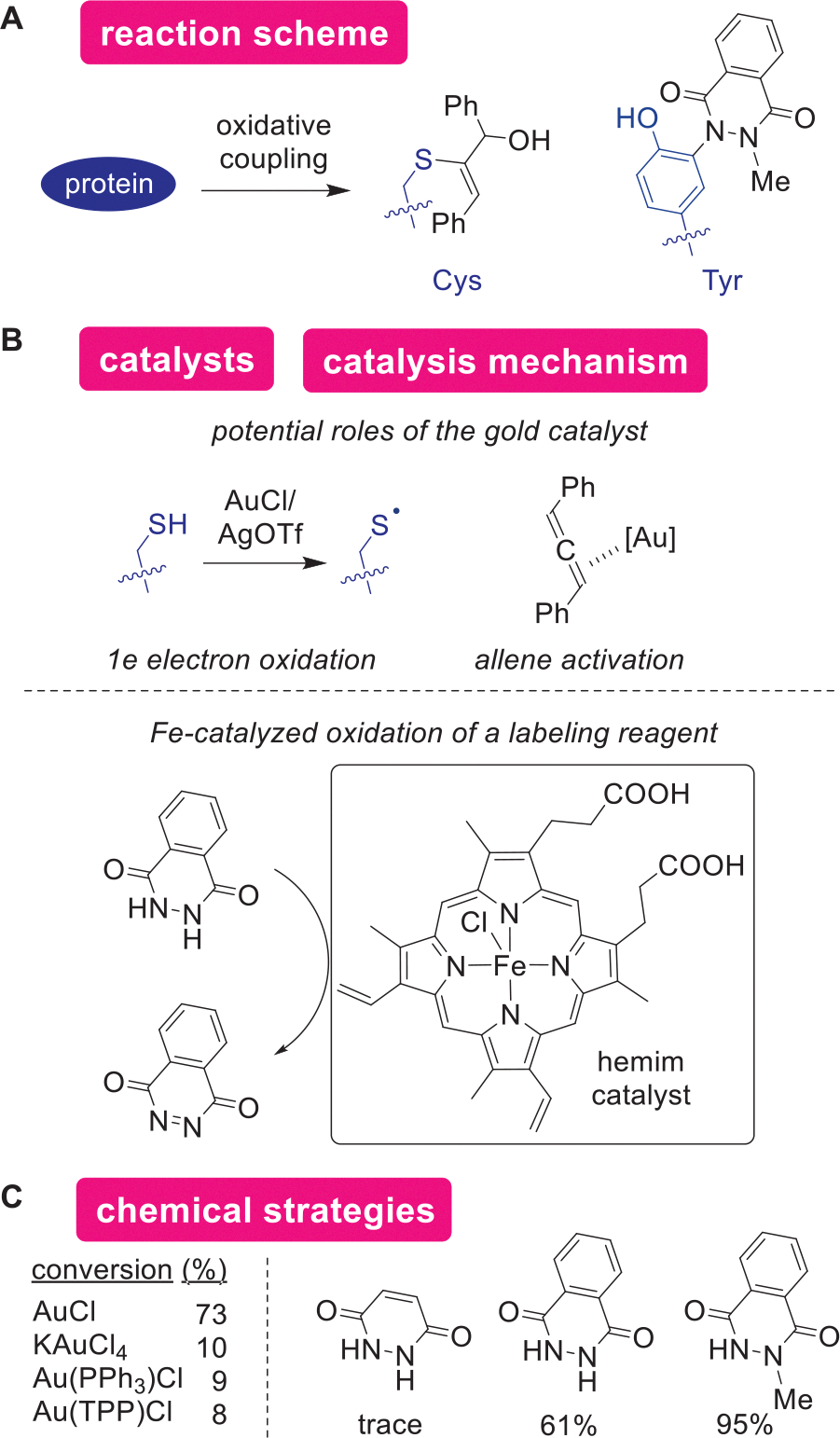
Metal-catalyzed oxidative coupling: (A) General reaction scheme. (B) Depiction of reaction promotion through oxidation of amino acid (top) or labeling reagent (bottom). (C) Effects of catalyst types and labeling reagents on the modification efficiency. TPP: tetraphenylporphyrin.

Catalytic oxidation can be applied for protein modification through activation of amino acid side chains and labeling reagents (Scheme 11A,B). As the oxidation of proteins is one of the fundamental processes in living systems, a range of oxidative reactions was used for protein modification in noncatalytic manners.^[161]^ The two major approaches for oxidative catalysis for protein modification occur through the activation of either amino acid side chains (e.g., thiyl radical generation) or labeling reagents (e.g., diazene generation). An example includes the alkenylation of a Cys residue by a gold- and silver-mediated system using allene-based labeling reagents.^[162]^ The gold catalyst was proposed to be useful for not only the single electron oxidation of Cys, but also the activation of the allene labeling reagent as a Lewis acid (Scheme 11C). The necessity of silver triflate additive may limit the utility of this catalysis, as a silver salt is known to induce precipitation of proteins (e.g., common staining protocols for protein gel known as silver staining).^[163]^

An oxidation process can be utilized for catalytic activation of labeling reagents, as the hemin-catalyzed Tyr modification takes advantage of such a mechanism.^[164]^ Inspired by biological oxidation processes of luminol in firefly chemiluminescence,^[165,166]^ this approach proceeds by the formation of Tyr-reactive diazene species through oxidation of the N–N bond to the N═N bond. A previous approach of Tyrselective modification by Barbas and coworkers necessitated the preparation of an unstable diazene reagent prior to protein modification processes,^[29]^ and this catalytic oxidation strategy omits the technical challenges through in situ generation of the active species. It is interesting that the most effective reagent proved to possess an *N*-methyl group that should not be able to form neutral N═N bond species as proposed (Scheme 11C). The high activity of the *N*-methyl reagent may be indicative of the potential involvement of single-electron oxidation by the iron complex.^[39]^

### 4.5. Catalytic Oxidative Cleavage

**Scheme 12.**
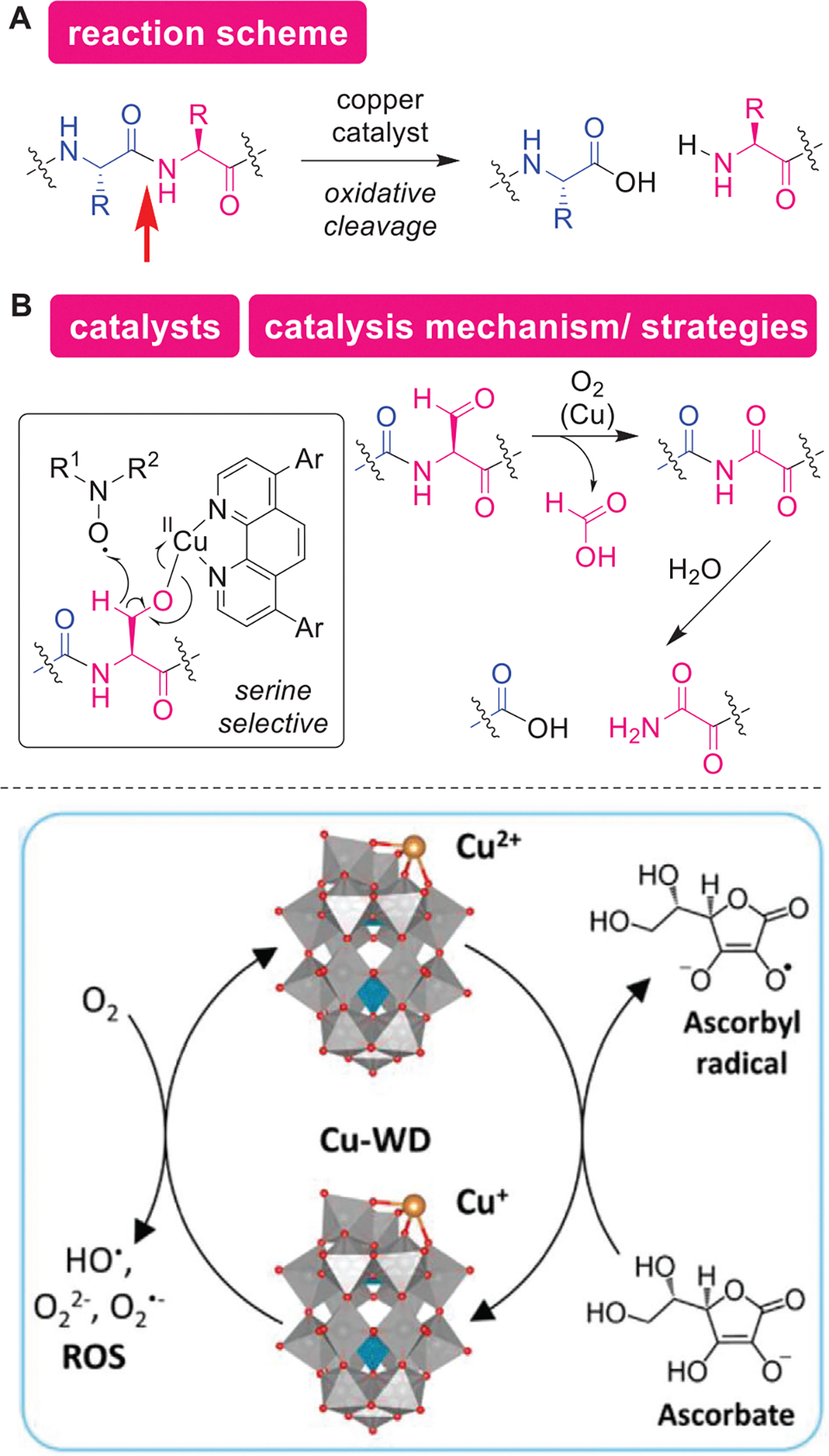
Copper-catalyzed oxidative cleavage of protein backbone: (A) General reaction scheme. The cleavage site is highlighted with a red arrow. (B) Ser-selective cleavage by a copper complex (top) and site-selective cleavage of lysozyme through reactive oxygen species (ROS) generation with a copper- and tungsten-based cluster (Cu-WD). The imide intermediate of the Ser-selective cleavage can be hydrolyzed at both imide C═O groups (blue and pink), but only one of the two possible hydrolysis products is shown for the sake of simplicity. The image of ROS-mediated cleavage was reprinted with permission.^[172]^ Copyright 2023, American Chemical Society (https://pubs.acs.org/doi/10.1021/jacsau.3c00011).

Oxidative cleavage of the peptide backbone can be induced through metal catalysis, mimicking enzymatic processes in natural systems (Scheme 12A). Reactive oxygen species (ROS) are important biological species both for physiological and pathological conditions, where protein oxidation plays pivotal roles.^[167,168]^ Through the sophisticated design of a catalytic system with a judicious choice of reaction components (catalysts, ligands, and oxidants), Oisaki and coworkers developed copper-mediated backbone cleavage, selectively at Ser residues (Scheme 12B).^[169]^ Single-electron oxidation of the primary alkylalcohol of Ser through copper(II)-phenathroline complex and *N*-oxide reagent initiates the catalysis, and subsequent oxidation of the generated aldehyde group produces a hydrolytically unstable imide intermediate that eventually undergoes the bond cleavage. Though minor reactions at Thr (secondary alkylalcohol) would occur, the Ser-selective cleavage was achieved even for a small protein, ubiquitin through this catalytic system. Another oxidative cleavage strategy is by a copper cluster that can generate ROS species with ascorbic acid to cause site-specific cleavage of lysozyme an enzyme (Scheme 12B).^[170]^ The 3D structure of the copper cluster was attributed to the observed site-specific cleavage of the enzyme through binding interaction between the cluster and enzyme. Similar ROS-mediated backbone cleavage was demonstrated by a vanadium cluster as well.^[171]^ As those reports showed a single example of a protein substrate for catalytic cleavage, the future directions of the field are likely to expand the generality and scope of the methods to achieve enzyme-like catalysis.

### 4.6. Lewis Acids

As acid-catalyzed processes are ubiquitous in enzyme active sites, numerous protein bioconjugation approaches have been also leveraged by a range of acid catalysts. In natural systems, enzymatic catalysis often depends on acid-mediated activation of weak electrophiles in proteins including proteolysis of amide backbones through interaction of acids to the carbonyl groups.^[173]^ While such enzymatic catalysis could often be substrate-specific processes, acid-catalyzed chemical modification of proteins can offer a broader substrate scope with a potentially unique reactivity and selectivity paradigm. One of the traditional approaches for acid-catalyzed protein modification is to employ strong Brønsted or Lewis acid (e.g., zirconium (IV) chloride-derived acid and perchloric acid) to enable reactions of weak electrophiles such as amides and carboxylic acids,^[174–177]^ although such harsh conditions may not be compatible with many protein substrates. More recently, various chemical strategies (e.g., sophisticated ligand design, proximity-accelerated catalysis through reversible covalent bond formation, and nonaqueous systems) have been devised to overcome the challenges, as described in the following sections. It is noteworthy that the development of many of those acid-catalyzed protein bioconjugation methods has been driven by knowledge of synthetic organic chemistry including Lewis acid strengths,^[127,128]^ metal affinity,^[178–180]^ and unique solvent properties.^[181]^ This section discusses catalysis by both Brønsted acid and Lewis acid including the metal Lewis acid

#### 4.6.1. Acid-Catalyzed Substitution Reaction

**Scheme 13.**
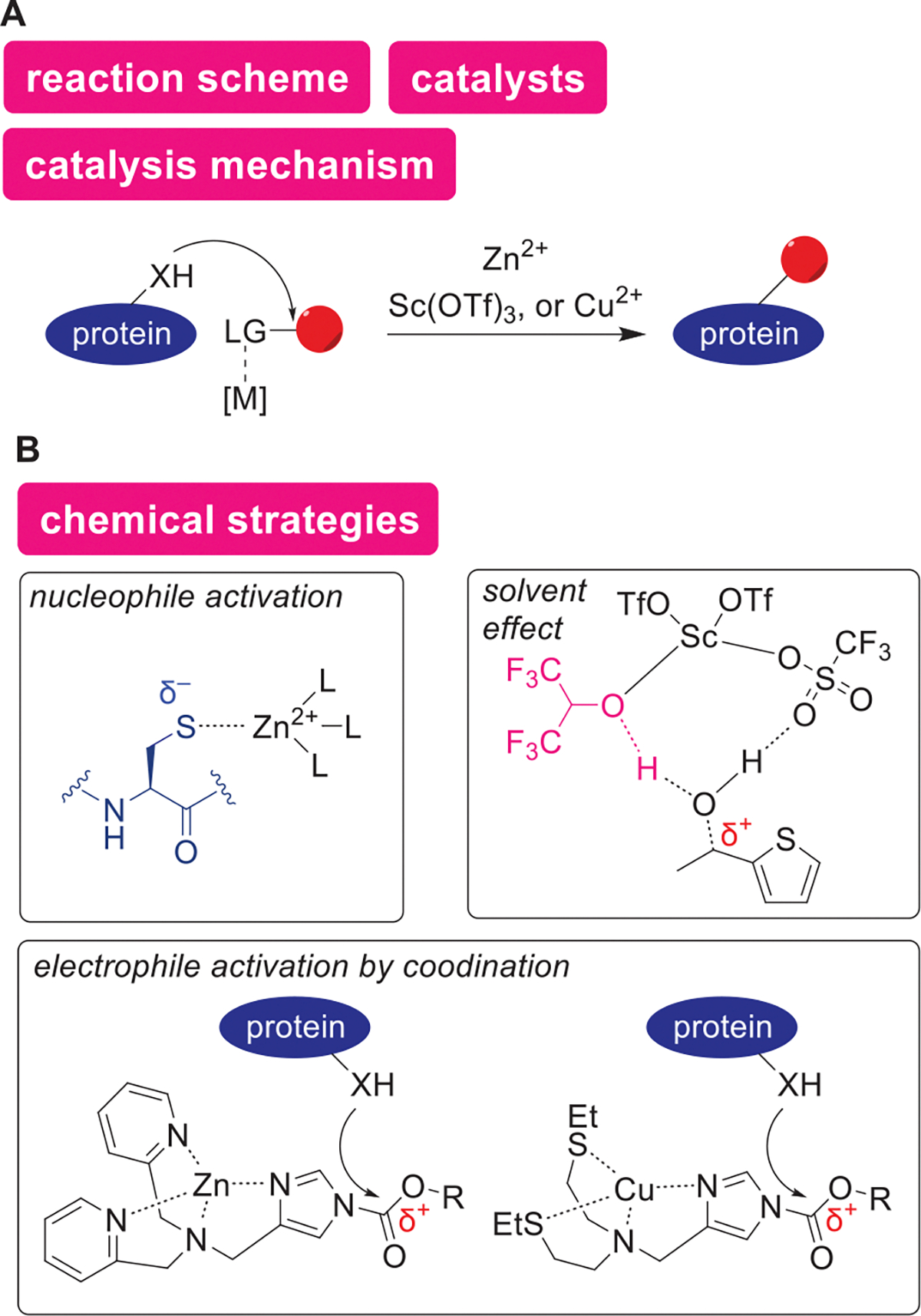
Acid-catalyzed substitution reactions: (A) Schematic illustrations of metal acid-catalyzed substitution reaction. XH: Nucleophilic side chains of amino acids such as Cys and Lys. LG: Leaving groups such as iodide, water, and imidazole derivatives. (B) Chemical strategies that enhance the catalytic substitution reactions. Top left: Activation of nucleophile by thiolate formation. Top right: Acid-catalyzed Friedel–Crafts reaction promoted by hexafluoroisopropanol (depicted in magenta). Bottom: Enhancement of electrophile reactivity through coordination to metal ions.

A zinc salt was employed as Lewis acid for lipidation of Cys through the catalytic activation of both nucleophiles and electrophiles (Scheme 13A,B).^[182]^ *S*-lipidation is a naturally occurring post-translational modification that is relevant to various cell signaling events including synaptic transmission and GPCR protein signaling.^[183]^ In the report by Fairlie and coworkers achieving a chemical way for *S*-lipidation, zinc ions played catalytic roles in the S_N_2 reaction between thiol groups on Cys residues and alkyl halides that contain fatty acid moieties. The zinc catalyst was suggested to have dual functions: increase of nucleophilicity of the thiol group and increase of electrophilicity of the alkyl halide. In other words, the catalyst would not only interact with the halide leaving group to enhance the electrophilicity of the alkyl halide, but the nucleophile of Cys residue can also be activated by the catalyst interaction lowering pKa of SH to thiolate ion. This design was perhaps inspired by a similar phenomenon found in natural zinc-containing enzymes (e.g., zinc-dependent transferases^12^ and zinc finger proteins^13^).^[184,185]^ As the catalysis proceeds with other divalent ions such as Ni^2+^ and Cd^2+^, the affinity of the metal ions to the thiol group might be playing key roles in the system.^[178–180]^

Hexafluoroisopropanol (HFIP) can facilitate electrophilic aromatic substitution of Trp through activation of thiophene–ethanol labeling reagent by a metal, Lewis acidic catalyst (Scheme 13A,B).^[186]^ HFIP, like other fluoroalcohols such as trifluoroethanol (TFE), is known to induce *α*-helical structures of the polypeptide,^[187–189]^ and protein substrates may not always tolerate such conditions.^[190,191]^ The recent work by Ohata and coworkers demonstrated that increased protein compatibility of HFIP by ionic liquid additives.^[186]^ For instance, an anti-HER2 antibody, trastuzumab was shown to lose its selective antigen-binding activity after treatment in HFIP, but the activity and selectivity were retained when the antibody was treated with HFIP-containing ionic liquids. Because HFIP and other fluoroalcohols are increasingly used for biomolecule modification recently,^[122]^ the potential compatibility of the solvents with proteins motivates their application for catalytic protein bioconjugation. The HFIP-based bioconjugation work by Ohata and coworkers took advantage of a Lewis-acid-catalyzed dehydrative alkylation reaction (Friedel–Crafts type process)^[192]^ of Trp residues.^[186]^ Catalytic actions of the Lewis acid such as scandium ions were studied using density functional theory (DFT) calculations, suggesting that the acidity of HFIP is increased through the coordination of HFIP to scandium. The increased acidity of HFIP by the coordination was shown to cause protonation and liberation of the OH group of the labeling reagent (thiophene–ethanol) as shown in Scheme 13B. In other words, the preliminary computational study indicated HFIP as a proton donor for the dehydration process, and consistent with the observation, the same group also demonstrated the use of Brønsted acid to catalyze the process as well.^[193]^ Although aqueous media have often been considered a requirement for useful protein bioconjugation methods,^[194]^ this example in addition to a carboxylic acid-based Ser modification method described below (the acid-catalyzed hydrolysis and alcoholysis section) may suggest practical usefulness of nonaqueous approaches. Another example of Trp modification in a nonaqueous solvent was recently reported by Otaka and coworkers.^[195]^ Modification of Trp of an antibody was achieved in an ionic liquid with 0.05% of TFA, a sulfoxide reagent, and magnesium chloride (MgCl_2_). The antigen-binding ability of the antibody was retained during the modification process, highlighting the effectiveness of the ionic liquid media for achieving large protein modification without denaturation.

Acyl imidazole derivatives have been applied as activity-based sensing probes to detect intracellular metal ions by leveraging the catalytic activity of the ions (Scheme 13A,B). Metal ions serve as chemical signals in cells,^[196,197]^ and chemical probes that detect these ions are useful for understanding their biological functions.^[198]^ In particular, reactivity-based sensing approaches offer advantages that traditional reversible binding sensors do not possess.^[199]^ An acyl transfer-based approach originally developed by Hamachi and coworkers relies on coordination of tetradentate acyl imidazole derivatives to metal ions (Lewis acid), which makes the carbonyl group more electrophilic to trigger a substitution reaction of the complex with nucleophiles on intracellular proteins.^[200]^ The original report developed a zinc-selective probe with dipicolylamine as the zinc-binding site. Although the binding affinity of the probe to Zn^2+^ was shown to be quite strong (0.7 nM) for this particular system, the approach could function as catalysis as long as there is a certain degree of dissociation process. Later, Chang and coworkers designed a copper-selective sensor through the incorporation of thioethers enabling the detection of labile brain copper.^[201]^ The same group recently applied the catalytic system to multiplex imaging of Cu(I) and Cu(II) by stimulated Raman scattering using isotopically labeled nitrile vibration tags.^[202]^ The development of the zinc- and copper-targeting acyl imidazole sensors indicates that ligand design would be able to modulate the binding selectivity to produce new catalytic chemical probes toward different intracellular metal ions.

#### 4.6.2. Acid-Catalyzed Hydrolysis and Alcoholysis

**Scheme 14.**
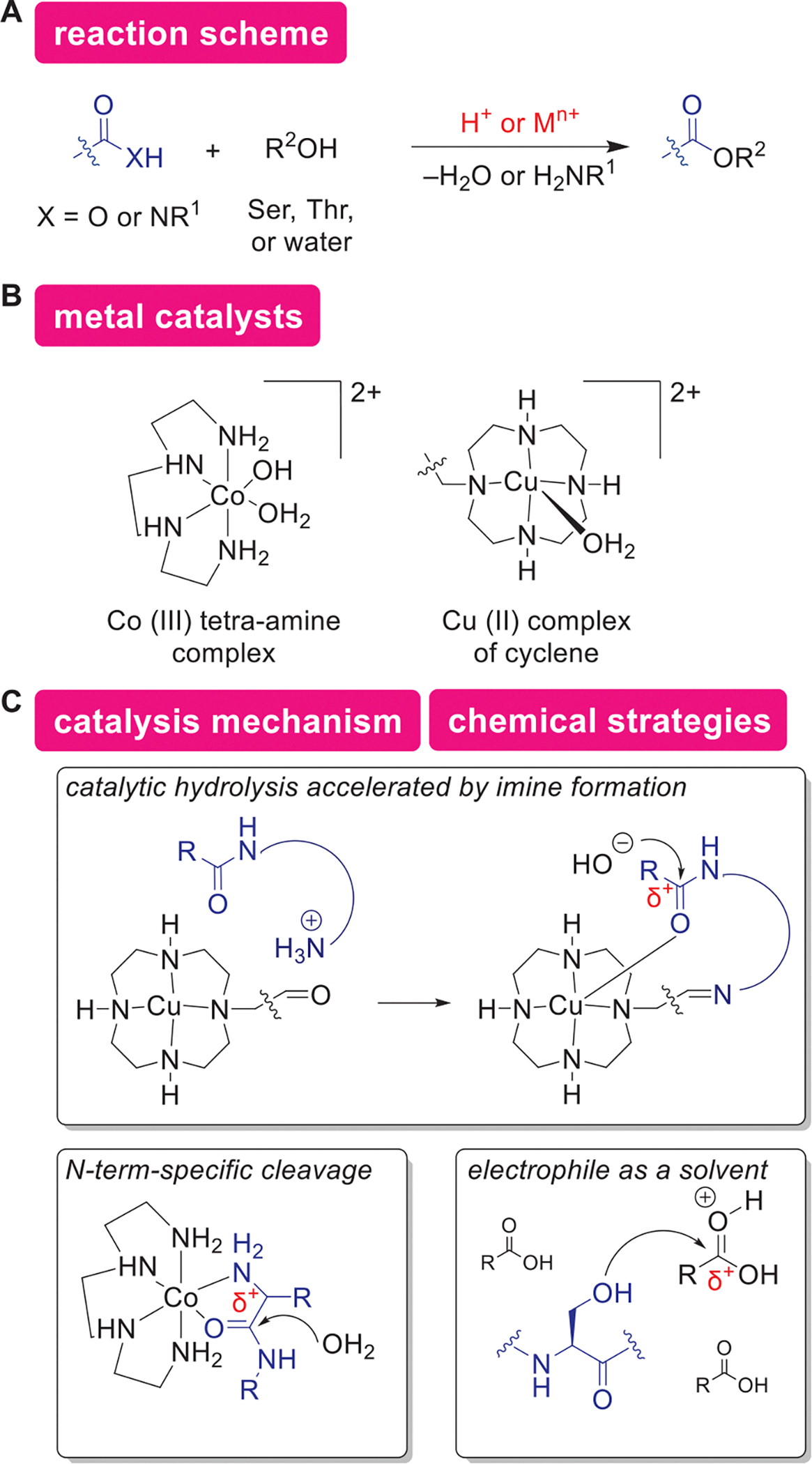
Acid-catalyzed hydrolysis and alcoholysis: (A) A general reaction scheme of acid-catalyzed hydrolysis/alcoholysis. (B) Structures of representative metal catalysts that mediate protein-backbone cleavage. (C) Chemical strategies that enhance the catalytic hydrolysis and alcoholysis. Top: Backbone hydrolysis accelerated by the copper center as Lewis acid and reversible formation of imine inducing proximity-driven effects. Bottom left: *N*-terminal specific cleavage by a CoIII catalyst. Bottom right: Catalytic acylation of alkylalcohols on proteins accelerated by an acid-activated carboxylic acid used as a solvent.

Lewis acidic metal complexes can serve as a catalyst to cleave amide bonds in proteins at specific amino acid sequences (Scheme 14A,B). Proteases such as trypsin or pepsin are enzymes to catalyze proteolysis (i.e., hydrolysis of the peptide backbones) and are widely used for proteomic studies.^[203]^ Although the proteolysis processes with such natural enzymes can proceed efficiently in mild conditions, their exclusive cleavage recognition patterns (e.g., Lys and Arg for trypsin) are not always compatible with a peptide or protein of interest. The development of non-natural, artificial proteolytic systems with alternative sequence recognition patterns has been actively studied.^[204,205]^ Metal catalysts that display Lewis acidic nature can be used as alternatives to natural proteases, for example. The metal center of such Lewis acid catalysts would facilitate the hydrolysis of the amide backbone by interaction with the carbonyl oxygen of the amide backbone making the carbonyl group more susceptible to the nucleophilic addition. An example includes a report by Kanai and coworkers showing scandium (III) triflate catalyst-mediated Ser/Thr selective cleavage. Kostic and coworkers reported a different type of amide backbone cleavage with His and Met selectivity using palladium-based catalysts.^[206,207]^ In addition, *N*-terminal residue-selective cleavage through the chelation of the *α*-amino group can be achieved with cobalt(III) complex as well (Scheme 14B,C).^[208]^ In order to facilitate selective and efficient cleavage processes by a metal complex, Suh and coworkers developed a system making use of reversible imine formation between aldehyde tethered to the metal–ligand and amine on proteins, which enabled specific cleavage at Gln(91)–Ser(92) and Ala(94)–Thr(95) of myoglobin protein (Scheme 14B,C).^[209]^ Such a reversible bond-forming process can be also applicable to the site-specific installation of functionality as well.^[210,211]^ Instead of reversible covalent-bond formation, supramolecular catalysis has been leveraged to induce proximity-driven effects for peptide cleavage as well.^[212]^ Those catalytic proteolysis examples demonstrated that choice of metals and ligand design can produce enzyme-like catalytic systems, and there have been many other reports of different metal systems as described in recent review articles.^[213–216]^

Ser residues in proteins can be catalytically modified in carboxylic acid-based non-aqueous media (Scheme 14C).^[217]^ Although Ser undergoes a variety of enzymatic modifications in living systems,^[218]^ chemical modification of Ser remains one of the challenging tasks owing to the modest nucleophilicity of the side chain and the abundance of the OH groups in aqueous media.^[219]^ Encourage by the catalytic Trp labeling in a nonaqueous medium (i.e., hexafluoroisopropanol or HFIP) as described above, Ohata and coworkers described that carboxylic acids could serve as a potentially protein-compatible reaction medium for Ser-targeting modification,^[217]^ which was motivated by the widespread use of many carboxylic acid-based compounds as biocompatible buffer components in biochemistry and protein science (e.g., acetate, glycine, and citrate buffers).^[220]^ The chemical modification of hydroxyl groups of Ser residue proceeds with an acid catalyst (e.g., trifluoroacetic acid and dysprosium(III) triflate) in carboxylic acid media where the excess carboxylic acid serves as an electrophile and reacts with the hydroxyl group (i.e., Fischer esterification-type reaction). The method was shown to be able to label protein substrates as well, including an intact antibody (trastuzumab), concanavalin A, and chymotrypsin. As chemoselective Ser labeling strategies have been simply lacking, this acylation chemistry may be indicative of the power of the nonaqueous, catalytic approach for protein modification

#### 4.6.3. Acid-Catalyzed Enolate-Based Reactions

**Scheme 15.**
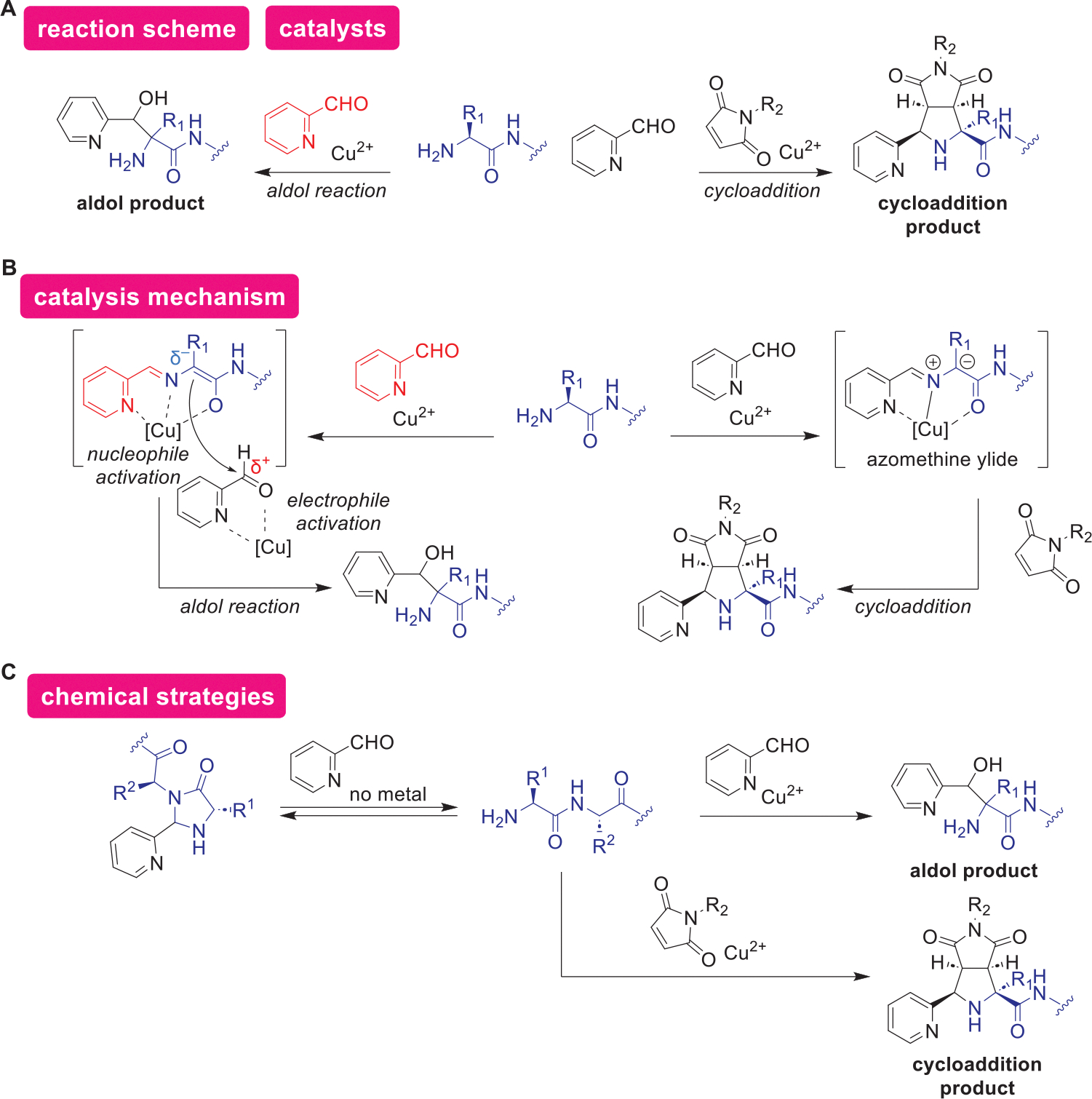
Dual catalytic aldol reaction (left) and copper-catalyzed cycloaddition (right): (A) Schematic illustrations of copper-/aldehyde-catalyzed aldol-reaction (left) and copper-catalyzed cycloaddition (right). The pyridyl-aldehyde acts as both the reagent and catalyst for the aldol reaction. (B) Catalysis mechanism of the copper-catalyzed aldol reaction (left) and copper-catalyzed cycloaddition (right). The pyridyl-aldehyde acting as a catalyst is depicted in red and the one as a regent in black. (C) Comparison of reaction products for *N*-terminal modification by choice of a labeling reagent and catalyst. Top: Alteration of the reaction product by the absence (reversible condensation reaction,^[226,227]^ left) and presence (Irreversible aldol reaction, right) of the copper catalyst. Bottom: Irreversible copper-catalyzed cycloaddition.

Aldol reactions using a dual catalytic system of copper and aldehyde catalysts enabled the labeling of protein *N*-termini (Scheme 15A). Site-specific protein labeling methods can be advantageous for producing well-defined protein conjugate compared to chemoselective approaches and are useful for various applications such as single-molecule localization microscopy,^[221]^ preparation of polyethylene glycol-tagged (PEGylated) therapeutic proteins,^[222]^ and production of antibody-drug conjugates.^[223]^ *N*-terminal amino groups can be attractive site-specific modification handles, as their decreased basicity compared to Lys amines can be utilized for *N*-terminal labeling through pH control.^[224]^ While there is a repertoire of *N*-terminal selective methods to date,^[224]^ Hanaya and coworkers reported a catalytic variant by utilization of copper and aldehyde catalysts activating *N*-terminal *α*-proton.^[225]^ The copper-catalyzed aldol reaction was proposed to occur through the activation of an *N*-terminal amino acid by the copper and aldehyde catalysts, forming a nucleophilic Cu(II)-enolate intermediate (Scheme 15B). This nucleophilic activation of the protein would be followed by electrophilic activation of another aldehyde molecule, eventually leading to aldol-type reactions between the activated species to produce the product with a stable C–C bond. Interestingly, in contrast to other acid catalysis earlier,^[186,217]^ other metal catalysts such as Sc(OTf)_3_ did not function as effective catalysts for the aldol reaction, perhaps indicating the importance of the subtle control of Lewis acidity and affinity toward certain ligands in this catalytic system. The aldehyde catalyst/reagent (2-pyridinecarboxaldehyde) was previously reported to form a hydrolytically unstable imidazolidinone product on *N*-termini of proteins,^[226,227]^ and the catalytic aldol process can be useful to produce more stable reaction products by alteration of the outcome of the aldehyde reaction with *N*-terminal amines by the introduction of the copper catalyst (Scheme 15C). Whereas, the hydrolytically unstable *N*-terminal product can be of use for reversible elimination of the chemical modification in a certain context,^[227]^ this catalytic example represents the importance to develop an alternative approach for production of protein conjugate with a different property. The same research group recently reported another copper(II)-mediated *N*-terminal modification by leveraging the hydrolytically stable intermediate (Scheme 15A).^[228]^ Kanemoto and coworkers reported copper-catalyzed [3+2] cycloaddition between metalated azomethine ylide (bidentate) on the Gly *N*-terminus of peptides and maleimides in organic solvents.^[229]^ Hanaya and coworkers expanded the scope of this approach to proteins by the use of pyridyl-aldehydes, allowing reactions in the aqueous buffer through the formation of a more stable intermediate, tridentate azomethine ylide (Scheme 15B,C). The reaction was applicable to peptides and proteins with various *N*-terminal amino acid residues. Site-specific modification of an antibody, trastuzumab with this modification method prepared antibody–drug conjugates with uniform drug–antibody ratio (DAR), which was applied to mice cancer models.

## 5. Organocatalysis

**Scheme 16.**
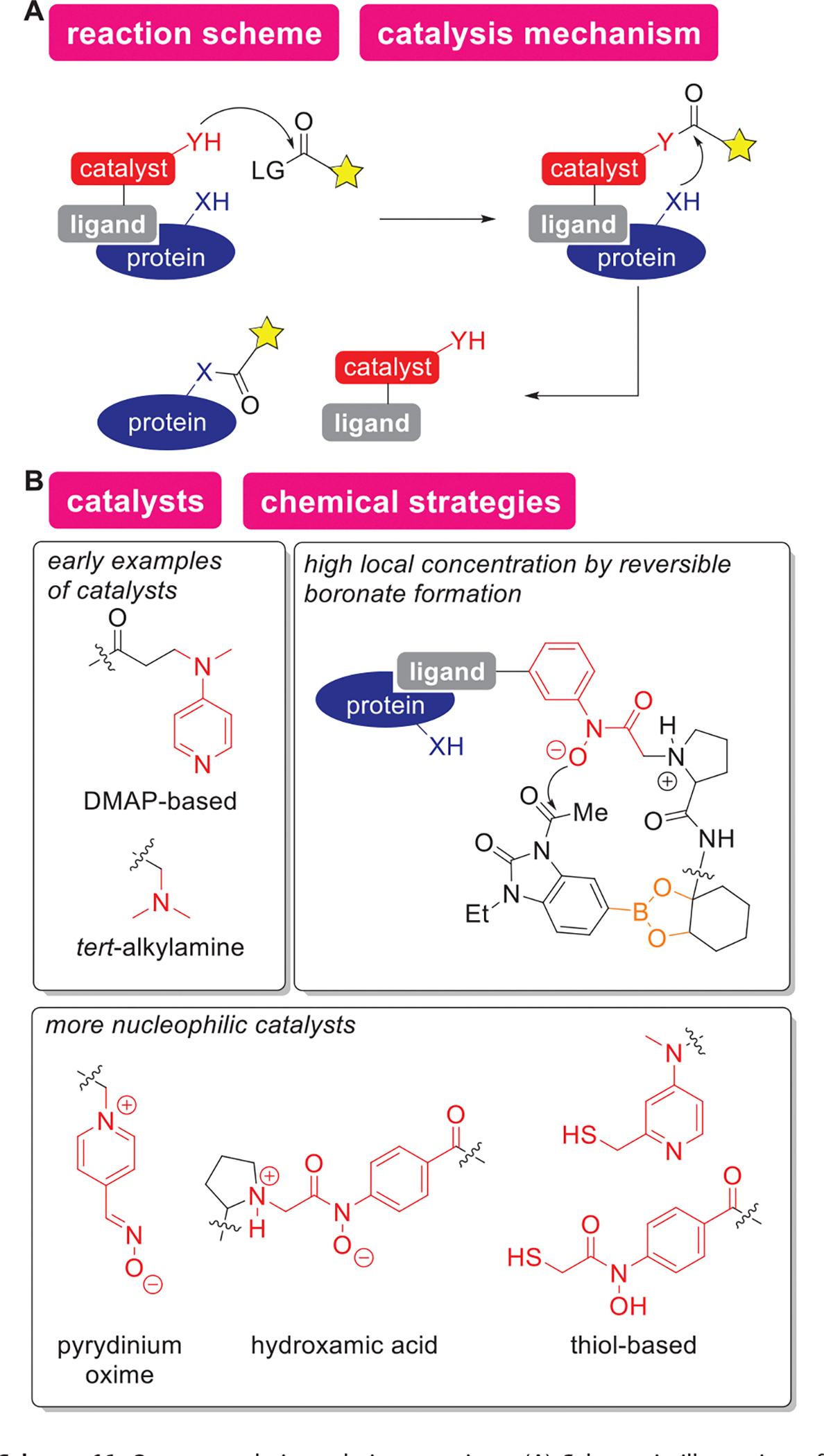
Organocatalytic acylation reactions: (A) Schematic illustration of organocatalytic acylation facilitated by the ligand-directed chemistry (i.e., binding of a pendant ligand to a protein binding pocket). (B) Representative catalysts and chemical strategies to enhance the organocatalysis. Top left: Chemical structures of early examples of the organocatalyst containing dialkylpyridine (DMAP: dimethylaminopyridine) or tertiary amine units. Top right: Catalysis enhanced by reversible boronate formation. Bottom: Modern strategies to increase catalytic activity/nucleophilicity of the organocatalysts including *N*-oxide with an inherent negative charge and thiol groups.

Organocatalytic protein bioconjugation has been achieved by modifying target proteins through organic ligand-assisted reactivity. Organocatalysis has been rapidly growing in the synthetic chemistry fields since the late 1990s.^[230]^ One of the major motivations for development of such catalytic strategies is to overcome the limitations of existing metal catalysts such as toxicity and high cost.^[231,232]^ For example, Pro is a readily available, nontoxic natural amino acid that serves as a catalyst for aldol reaction and Mannich reaction.^[233]^ Even though a wide variety of organocatalytic transformation has been reported for small molecule substrates decades after its inception,^[234–237]^ it is interesting that protein bioconjugation driven by organocatalysis has been exclusively by acyl transfer reactions through proximity-accelerated chemistry to date. As also described in the supramolecular catalysis section, such proximity-driven chemistry functions through association and dissociation of an affinity ligand that interacts with a protein of interests. The reported organocatalytic bioconjugation processes generally proceed in two steps (Scheme 16A): (1) association of a ligand to a target protein accelerates nucleophilic attack of ligand-tethered nucleophilic catalyst to an acyl donor reagent and (2) another set of nucleophilic attacks by proteins to the activated acyl donor occurs, followed by dissociation of the ligand–catalyst. The following paragraphs focus on evolution of acyl transfer-based organocatalytic bioconjugation methods and brief demonstration of their utilities and applications

### 5.1. Organocatalytic Acylation

Even in the first-generation labeling systems, organocatalytic acylation reactions through protein–ligand interaction demonstrated their usefulness for site-specific modification of target proteins for live cells and tissue samples (Scheme 16A). The seminal work of the organocatalytic bioconjugation was by Hamachi and coworkers, which demonstrated acylation of a glycoprotein-binding protein, lectin with a dialkylaminopyridine-based catalyst (i.e., dimethylaminopyridine or DMAP-type catalyst, Scheme 16B).^[238]^ The DMAP catalyst was tethered with saccharide–ligand, and thiophenyl esters were employed as acyl donors for the site-specific labeling of lectins. By the virtue of proximity-driven effects, target proteins can be selectively modified even in the presence of other proteins (e.g., in cell and tissue lysates). In addition to saccharide ligands, the DMAP-based catalyst can be conjugated to proteins that bind to protein targets; for instance, DMAP-tethered lectin was utilized for the labeling of glycoproteins on live cell surfaces.^[239]^ As a similar approach, a DMAP-tethered antibody fragment was developed to selectively modify receptors on cell membranes, which enabled epitope mapping of antibodies.^[240]^ Another early example of ligand-directed catalysts is an organocatalyst based on dimethylalkylamine tethered with biotin, which was used for modification of carboxyl groups of Asp and Glu residues of avidin.^[241]^

More efficient catalysis at physiological pH than the first-generation DMAP-based catalysis was demonstrated with anionic catalysts and thiol-based catalysts with milder acyl donors (Scheme 16B).^[242–244]^ One of the challenges in the DMAP catalysis is that p*K*_a_ of the conjugate acid of the DMAPbased moiety is 8.6,^[243]^ and substantial portions of the catalyst could be protonated at physiological pH ranges. To this end, pyrydinium oximes and hydroxamic acids were proposed as alternatives because of their lower p*K*_a_s (6.6^[245]^ and 6.5,^[243]^ respectively) that could allow faster protein labeling than DMAP at physiological pH. Thiol-based catalysts possess additional benefits, compared to DMAP-based catalysts, that are kinetically favorable thiol–thioester exchange between the catalyst and the acyl donor.^[246–250]^ As an independent approach from the thiol-based catalysis, Kanai and coworkers developed a unique proximity-driven approach through reversible boronate formation to facilitate the organocatalytic modification for site-specific modification of target within live cells (Scheme 16B).^[244]^ Such a range of the second-generation catalysts allowed the use of moderate electrophilic acyl donors (e.g., acetyl-CoA) compared to the one used with DMAP-based catalysts (thioesters derived from thiophenol), attenuating off-target labeling.^[242,248]^ For example, in the DMAP-based catalysis, the reaction usually requires a low temperature (e.g., 4 °C) to minimize the non-specific labeling arising from the high electrophilicity of the thioester acyl donor.^[242]^ Therefore, the use of milder acyl donors such as *N*-acyl-*N*-alkyl sulfonamides,^[242]^ alkyl thioesters,^[246–250]^ and acyl imidazoles^[251]^ can be beneficial through suppression of the labeling agents’ off-target reactivities. A notable application of the second-generation approach is that a hydroxamic acid-thiol-based catalyst conjugated with histone-ligand has been used for acylation of Lys-120 (K120) of histone H2B with endogenous acyl-CoA as an acyl donor.^[250]^ Since acetylation of Lys residues of histone proteins is a naturally occurring post-translational modification that regulates gene expression (epigenetic regulation),^[252]^ this approach could be useful for the chemical manipulation of the epigenetic regulation of gene expression.

## 6. Supramolecular Catalysis

**Scheme 17.**
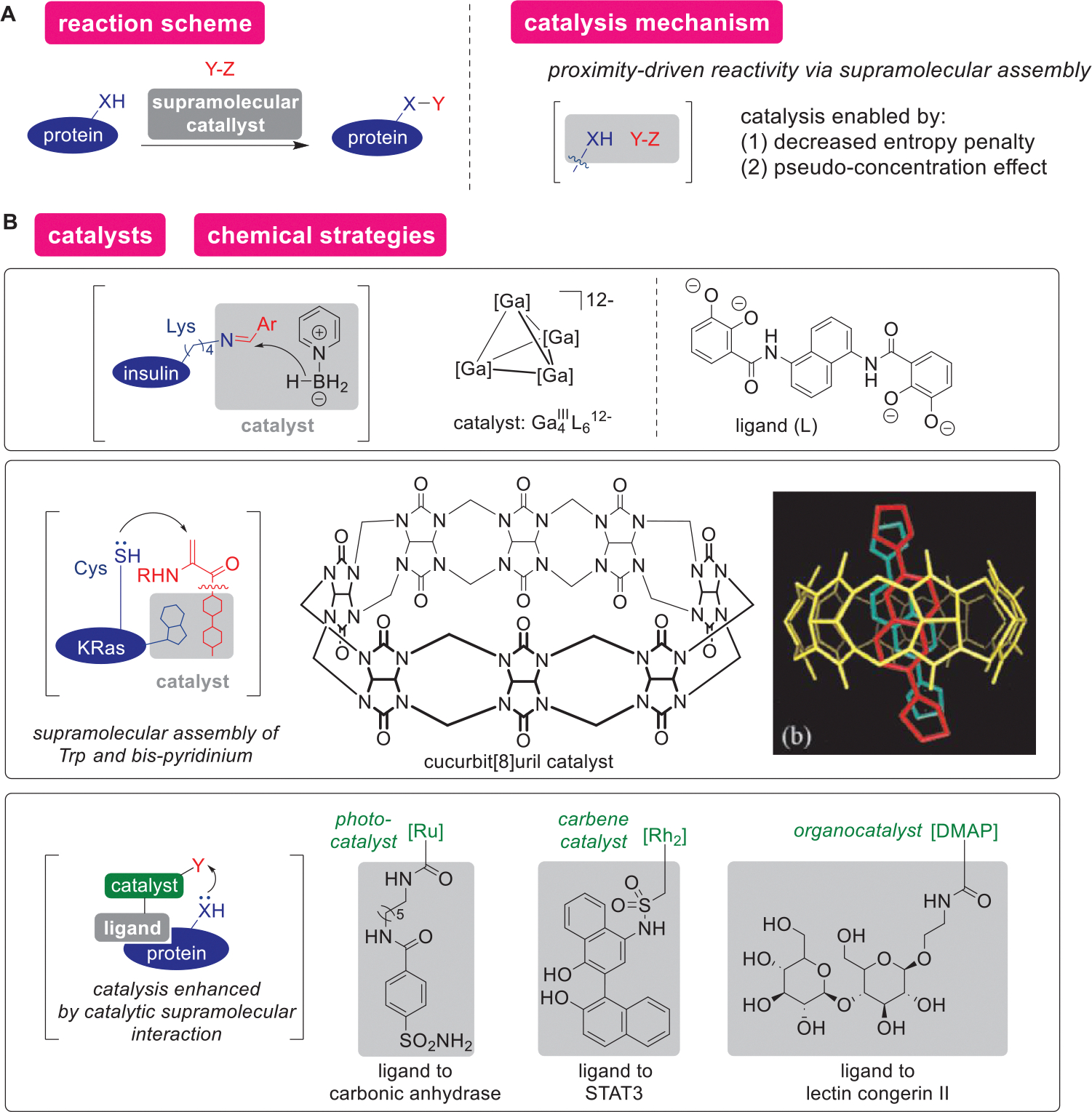
Supramolecular catalysis: (A) Schematic illustration and mechanism of the protein modification with supramolecular catalysts. (B) Structure of supramolecular catalysts and catalysis mechanisms. Top: Reductive amination catalyzed through the supramolecular interaction between the gallium cluster, pyridine-borane, and imine intermediate. Middle: Thia-Michael addition to a Cys residue facilitated by the interaction between a Trp residue of a protein and bipyridinium unit of the labeling reagent in the cucurbit[8]uril (CB[8]) as a supramolecular host. The image of X-ray crystal structure on the right was reproduced with permission.^[262]^ Copyright 2000, American Chemical Society. Bottom: Proximity-driven catalysis through binding of a ligand to target proteins.

Supramolecular chemistry-based strategies such as metal-anion interactions, macrocyclic self-assembly, and ligand-directed affinity labeling can promote proximity-induced reactivities for protein bioconjugation (Scheme 17A). Supramolecular chemistry pertains to molecular assembly through a range of noncovalent interactions.^[253]^ The process can be viewed as host–guest chemistry that causes a covalent bond-forming reaction between a substrate and reagent through supramolecular forces including hydrophobic interaction, hydrogen bond, van der Waals force, *π–π* stacking, and ion–dipole effect.^[254]^ Enzymatic systems make use of a number of types of supramolecular chemistry to achieve site-selective modification. For example, a transpeptidase sortase utilizes its domain called *β*6/*β*7 loop that recognizes an LPXTG (X = D, E, A, N, Q, or K) motif of a target peptide/proteins for site-selective modification of their C-terminal positions.^[255,256]^ Supramolecular chemistry has been used for noncatalytic bioconjugation reactions including metal-anion ionic interactions by His_6_-tag/Ni system and DNA/RNA hybridization through base pairings, as such non-catalytic supramolecular bioconjugation reactions have been reviewed in a recent article.^[257]^ As described below, artificial host molecules such as gallium cluster and cucurbituril as well as ligand-directed affinity labeling strategy (see the organocatalyst section) were used as catalytic protein labeling strategies

### 6.1. Reductive Amination Through Host–Guest Chemistry

Lys-selective reductive amination can be achieved catalytically by a gallium cluster host (Scheme 17B).^[258]^ For small molecule substrates, a gallium cluster Ga_4_L_6_ (L = *N*,*N′*-bis(2,3-dihydroxybenzoyl)-1,5-diaminonaphthalene) host was known to cause catalytic Nazarov cyclization and aza-Darzens reactions through the assembly of reaction components by hydrophobic effect and stabilization of cationic intermediates (i.e., electrostatic stabilization of cations by the polycationic host Ga_4_L_6_).^[259,260]^ Even for peptide and protein bioconjugation purposes, the same anionic gallium cluster can serve as a supramolecular host to catalyze reductive amination reactions on alkylamine groups of Lys residues. The specificity to Lys residues for the supramolecular catalysis contrasts with traditional reductive amination with a borohydride reagent (e.g., NaCNBH_3_), which often cannot differentiate Lys and *N*-terminal amines unless there is precise pH control.^[261]^ The selectivity mechanism was not studied in this report, but the supramolecular host catalyst’s preference toward sterically more accessible *ε*-amine may be the reason for the observed phenomena.

### 6.2. Michael Addition Through Host–Guest Chemistry

A macropolycyclic catalyst, cucurbit[8]uril can induce proximity-driven Michael addition through the assembly of a Trp residue and bipyridinium derivative (Scheme 17B). Cucurbituril is a macropolycyclic compound assembled from glycoluril and formaldehyde.^[262]^ Cucurbituril acts as a host molecule for various guests such as hydrocarbons, saccharides, dyes, amino acids, and proteins through hydrophobic interactions, ion–dipole interactions, and dipole–dipole interactions.^[263]^ Through such supramolecular host capability, cucurbituril-mediated chemistry has been employed for protein modification such as azide-alkyne cycloaddition reactions enhanced through the hydrogen bonding network.^[264]^ For modification of proteins with natural amino acid side chains, cucurbit[8]uril—a cucurbituril that contains eight glycoluril units—was used for supramolecular catalysis-based Michael addition reaction modifying a Cys residue through inclusion of both a bipyridinium group on the labeling reagent and a Trp residue simultaneously.^[265]^ Through this approach, cucurbit[8]uril-facilitated modification of a KRas protein was achieved using dehydroalanine–bipyridinium reagent; cucurbit[8]uril served as a host to a Trp residue of KRas and bipyridinium for labeling, followed by a proximity-induced thia-Michael reaction between Cys residue on the substrate and dehydroalanine on the labeling reagent. It should be also noted that supramolecular catalysis-based chemical backbone hydrolysis has been also achieved using a polymacrocyclic catalyst, although their substrate scope is limited to peptides.^[212]^

### 6.3. Ligand-Directed Labeling

Protein–ligand interactions have been utilized for a variety of catalytic transformation by enhancing inherently slow reactions through proximity-driven effects (Scheme 17B). Early examples of ligand-directed labeling strategies (noncatalytic) were shown to modify active sites of enzymes and antibody binding sites.^[266,267]^ Such early examples were not catalytic, as the bound ligand does not dissociate after the labeling process. More recently, catalytic ligand-directed strategies have been reported where types of ligands range from small molecules to peptide ligands, and various catalysis, as described in the previous sections (i.e., photocatalysis, transition-metal catalysis, and organocatalysis). The proximity effect is often utilized for sluggish reactions that do not proceed without the rate enhancement mechanism, including examples of ruthenium photocatalysis and DMAP-based organocatalysis.^[60,238]^ In other words, generally unreactive amino acid residues could be modified through the proximity effects, as modification of Phe was achieved by dirhodium catalyst conjugated with STAT3 ligand.^[268]^ The proximity-driven rhodium catalysis was indeed shown to be capable of modification of half of canonical amino acids, including Asn, Phe, Gln, and Thr,^[269]^ showcasing the power of the supramolecular chemistry.

## 7. Heterogeneous Catalysis

**Scheme 18.**
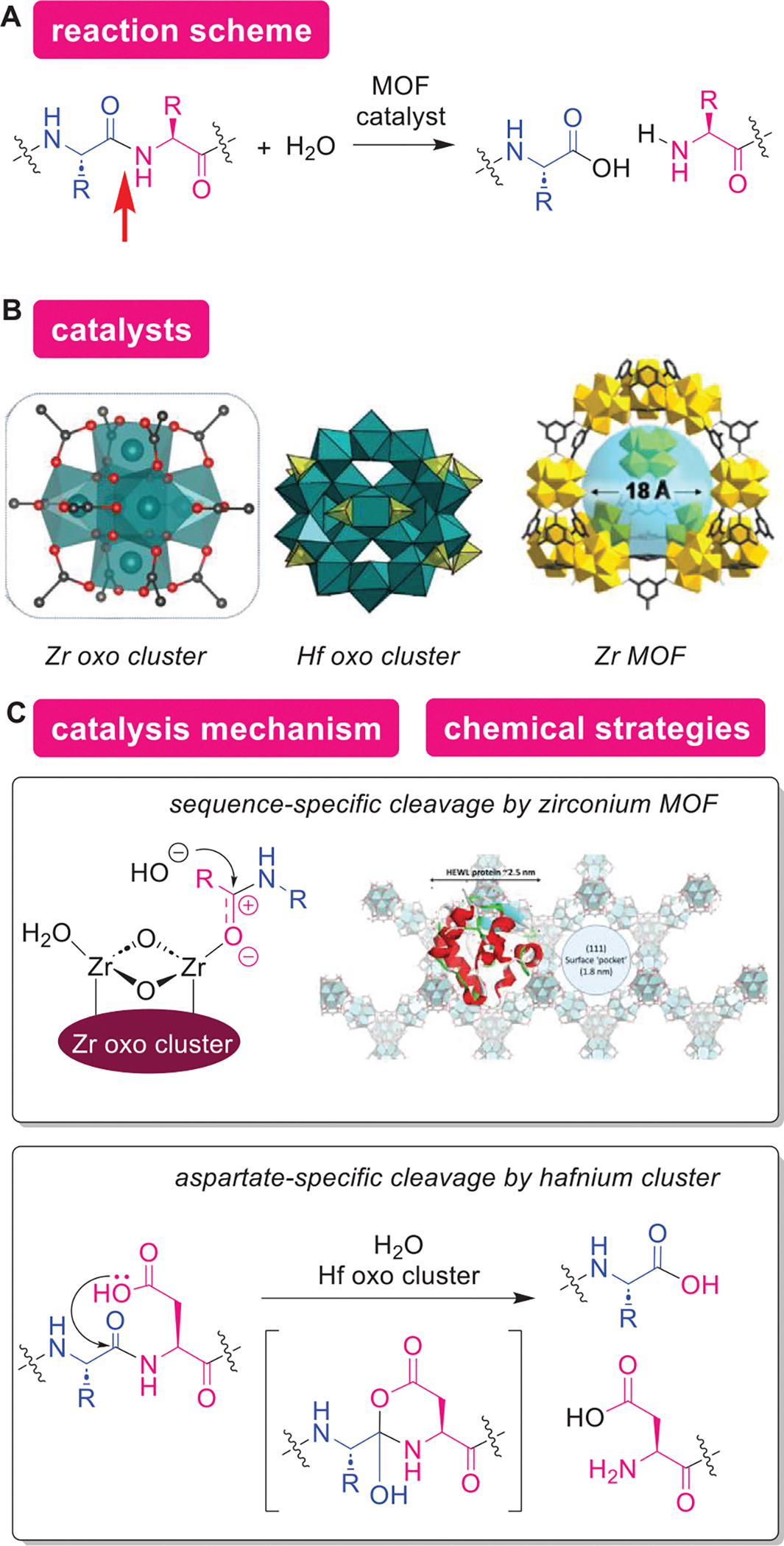
Heterogeneous catalytic hydrolysis: (A) Schematic illustrations of hydrolysis of the peptide backbone catalyzed by metal clusters and metal-organic framework (MOF) catalysts. (B) Structures of representative heterogeneous and related catalysts. The image of Zr oxo cluster was reprinted with permission.^[285]^ Copyright 2024, American Chemical Society. The image of Hf oxo cluster was reprinted with permission.^[292]^ Copyright 2020, Wiley-VCH Verlag GmbH & Co. KGaA, Weinheim. The image of Zr MOF was reprinted with permission.^[284]^ Copyright 2018, American Chemical Society. (C) Chemical strategies that enhance peptide hydrolysis by MOF and oxo cluster catalysts. Top: size comparison of lysozyme and pore-size of the zirconium MOF, and hydrolysis catalyzed by zirconium oxo-cluster. Bottom: sequence-specific hydrolysis via cyclic hemiaminal intermediate by hafnium cluster. The image of Zr MOF and protein was reprinted with permission.^[284]^ Copyright 2018, American Chemical Society.

Heterogeneous catalysts could be beneficial for proteolysis applications due to their tunable properties and facile separation from products (Scheme 18A). One of the earliest documented heterogeneous catalysis is Faraday’s oxidation reactions by platinum catalysts in the 1800s.^[270]^ The advantages of heterogeneous catalysts in comparison to homogeneous catalysts are reusability and easier separation of catalysts.^[271]^ These advantages are especially beneficial in the industrial large-scale processes.^[272]^ There are many heterogeneous catalytic systems for small molecule substrates,^[273–275]^ and recently, His functionalization of peptides with a heterogeneous catalyst was reported.^[276]^ However, catalysts that can be applicable for protein substrates are quite scarce.^[277]^ As described in the following paragraphs, metal-organic framework (MOF)-based platforms are one of the few examples that act as heterogeneous catalysis for protein substrates. MOFs are crystalline materials composed of metal ions and organic ligands. Properties of MOFs such as pore size, type of metal ions, and surface area are tunable.^[278,279]^ As described in the acid catalyst section, artificial proteases composed of non-biomolecule building blocks could be useful for the digestion of proteins because of unique cleavage sites of such approaches. While MOF catalysts are also useful for the same purposes (i.e., hydrolysis of the protein backbones), the protein-hosting ability of MOFs can be an additional advantage for the following two reasons:^[277]^ (1) the space confinement effect of MOF mesopores could mimic enzymes active site, as demonstrated in a few reports^[280,281]^ and (2) the heterogeneous nature of MOFs can be advantageous for proteomics digestion purposes, as the catalysts can easily be separated from products after the reaction. As free metal ions could also be a catalyst for given reaction systems,^[215]^ it is often important that a MOF catalyst possesses chemical and structural stability, so that fragments/components of MOF through decomposition would not induce unwanted processes.^[282,283]^ For instance, one of the early reports for MOF-catalyzed proteolysis described leaching of Cu(II) ions from the MOF,^[277]^ although the Cu(II) ion was not ascribed to the catalytic activity of the system.

Zirconium-based MOF and metal-oxo clusters were demonstrated to mediate backbone cleavage of proteins through their Lewis acidic actions (Scheme 18B,C). ParacVogt and coworkers introduced a zirconium(IV)-based MOF as a heterogeneous catalyst for proteolysis of model protein substrates.^[284]^ The hydrolysis was proposed to proceed through the activation of the amide backbones by Lewis acidic Zr(IV) centers incorporated in the MOF catalyst. Hexazirconium metal-oxo cluster was a building block of the specific MOF catalyst, which was assembled by capping and interconnection by six benzene-1,3,5-tricarboxylate linker in a trigonal antiprism fashion. The water-soluble metal-oxo cluster, [Zr_6_O_4_(OH)_4_(CH_3_CO_2_)_8_(H_2_O)_2_Cl_3_]^+^ showed superior hydrolytic activity compared to the zirconium MOF catalyst.^[285]^ Some metal-oxo cluster-based catalysts (e.g., cerium-based polyoxometalate^[286]^ and molybdenum-based polyoxometalate^[287]^) showed unique properties such as regioselectivity potentially arising from the enzyme-like noncovalent interaction with specific regions of proteins.^[288,289]^ Another explanation for such enzymatic behaviors is that the catalysts might make cleavage sites more accessible through partial unfolding of protein structures of proteins.^[286,290,291]^ As the hexazirconium metal-oxo cluster is a component of the MOFs,^[285]^ the metal-oxo cluster component has been used for mechanistic studies showing the accessibility of the catalyst active site by protein substrates and its similarity of the catalytic action to the hafnium cluster as described below.

Multinuclear hafnium metal-oxo cluster, [Hf_18_O_10_(OH)_26_(SO_4_)_13_·(H_2_O)_33_], would be an effective heterogeneous catalyst for proteolysis specifically at Asps (Scheme 18B,C).^[292]^ there have been several reports of Hf(IV)-based MOFs as heterogeneous catalysts for organic reactions that involve activation of carbonyl groups.^[293,294]^ The Hf_18_ polynuclear cluster [Hf_18_O_10_(OH)_26_(SO_4_)_13_·(H_2_O)_33_] is insoluble in water and possesses both Lewis and Brønsted acidic moieties (i.e., protic protons of the coordinated water on the Hf centers) that was shown to facilitate hydrolysis of the amide backbones of a protein.^[292]^ The catalysis displayed selective cleavage at Asps (both Asp–Xxx and Xxx–Asp bonds where, Xxx is an arbitrary amino acid residue), and the proposed reaction mechanism is by nucleophilic attack of the Asp COOH to the amide backbone that forms an anhydride intermediate for Xxx–Asp bond cleavage and an imide intermediate for Asp–Xxx bond cleavage, followed by another set of nucleophilic attack by a water molecule to complete the process. It should be noted that presumably due to the Brønsted acidity of the catalyst, negatively charged regions of proteins were effectively cleaved, which was not achieved with the Zr(IV)-based metal-oxo cluster catalyst.^[288,292,295]^ Therefore, hafnium-based catalysis may offer an alternative selectivity for proteolysis applications.

## 8. Summary and Outlook

Catalytic transformations have shown the great utility in chemical modification of proteins from various viewpoints, and the compilation and analysis of a set of literature in this review also underscored possible future directions of the realm of the catalytic bioconjugation research. Appearance of redox-mediated chemistry in a multitude of sections is notable, probably implying its growing interests across various catalysis fields. However, because oxidation and reduction reactions are common processes in several canonical amino acid side chains,^[296]^ strategies to suppress unwanted side reactions may be the unavoidable tasks as literature precedents also tackled the issue already (e.g., reactions under inert atmosphere^[297]^ and use of redox-sensitive additives^[298]^). It became also obvious that some catalytic strategies are limited to only a certain reaction type or virtually nonexistent for protein bioconjugation purposes. For instance, organocatalysis and heterogeneous catalysis have been realized only through acyl-transfer reactions and backbone hydrolysis, respectively. We were unable to find any examples of asymmetric catalysis and mechanochemical catalysis that have been used for bioconjugation at a protein level, though potential usefulness of some reactions for small molecule substrates in this areas are indicated in recent literature.^[299–301]^ In particular, the dearth of the asymmetric catalysis is striking given the enantiomeric/geometric importance of post-translational modification in nature (e.g., Met oxidation^[302]^ and Lys acetylation^[303]^) as well as increasing studies on *d*-amino acid/*d*-proteins.^[304]^ Dehydroalanine functionalization would be an example highlighting this trend, as there have not been examples of protein bioconjugation utilizing dehydroalanine in an asymmetric fashion^[157,305]^ even if a plethora of reports demonstrated asymmetric conjugate addition reactions at a small molecule level.^[306]^ Plausibly, this challenge may have been exacerbated by limited availability of analytical techniques that can be usable to differentiate the isomer forms of a particular amino acid residue in protein substrates. The examination of literature also showed the power of dual catalytic systems, especially those combined with supramolecular catalysis for site-specific or target-specific labeling strategies. Indeed, hybrid catalytic systems have been frequently utilized in chemistry of small molecules and peptides,^[307]^ and protein bioconjugation may benefit from such hybrid systems as well. Finally, it is noteworthy that some catalytic mechanisms would be only possible in polypeptide substrates but not simple small molecule substrates (e.g., copper-catalyzed backbone modification of amide N–H driven by a neighboring His residue^[146]^), and proteins may serve as a platform to expand the boundary of the catalysis domain beyond the small molecule chemistry. Diverse fields spanning bioorganic chemistry, chemical biology, biomedical science, and material science necessitate development of protein bioconjugation to address various scientific and pragmatic challenges, and the chemical strategies and principles mentioned in this review paper may be a catalyst to transcend the limit.

## Supplementary Material

supplementary material

Supporting Information

The list of cited protein modification papers is in the Supporting Information as an Excel spreadsheet.

Supporting information for this article is available on the WWW under https://doi.org/10.1002/cctc.202402125

## Figures and Tables

**Table 1.** Overall advantages and disadvantages of catalytic protein bioconjugation methods described in this review.

	Advantages	Disadvantages

**Electrocatalytic bioconjugation**	• Virtually no efforts to remove a catalyst from solution (e.g., electrons and electrodes). • Facile process to turn on/off reactions.	• Irreversible adsorption of proteins to electrodes. • Tendency to cause unwanted redox-based side reactions (e.g., side chain oxidation). • Necessity of specialized reaction equipment/setup.
**Photocatalytic bioconjugation**	• Facile process to turn on/off reactions. • Applicability of the catalysis in cellular contexts.	• Necessity of inert atmosphere during reactions to suppress oxidative side reactions.
**Metal-catalyzed bioconjugation**	• Modular control of reactivity and selectivity by ligand design. • Applicability of the catalysis for detection of cellular metal ions. • Availability of numerous types of metals and ligands.	• Generally high toxicity of metals for cellular samples. • Known protein precipitation/denaturation by metals.
**Organocatalytic bioconjugation**	• Biologically benign components in a catalyst. • Applicability of the catalysis in cellular contexts.	• Requirement of proximity-driven effects (i.e., no examples of chemoselective organocatalytic bioconjugation to date). • Limited catalysis types (e.g., acyl transfer).
**Supramolecular catalysis-based bioconjugation**	• Realization of target-specific modification even in a complex mixture such as cellular samples. • Realization of site-specific modification beyond chemoselective labeling. • Applicability of the catalysis in cellular contexts.	• Requirement of target-specific design of a catalyst/ligand (i.e., a lack of generality compared to chemoselective methods).
**Heterogeneous catalysis-based bioconjugation**	• Facile removal of a catalyst after reactions.	• Irreversible adsorption of proteins to the catalyst. Limited catalysis types (i.e., proteolysis).

**Table 2.** Medium redox potentials of redox active amino acid residues.

Residues	Experimental *E*_m7_

Tyr	0.91
Trp	1.03
His	1.17
Cys	0.093
Met	1.2–1.5
Lys	1.24

## Data Availability

The data that support the findings of this study are available in the Supporting Information of this article.
